# Inherited regulation for advanced ARTs: comparing jurisdictions’ applications of existing governance regimes to emerging reproductive technologies

**DOI:** 10.1093/jlb/lsab034

**Published:** 2022-02-04

**Authors:** Walter G Johnson, Diana M Bowman

**Affiliations:** Sandra Day O’Connor College of Law, Arizona State University, 111 E Taylor Street, Phoenix, AZ 85004, USA

**Keywords:** Assisted reproductive technologies, mitochondrial replacement therapies, inherited regulation, governance, emerging technologies

## Abstract

Over the past 5 years, advanced assisted reproductive technologies (ARTs), such as mitochondrial replacement therapies (MRTs) and heritable human genome editing (HHGE), have raised global policy concerns and fears of ‘unregulated’ proliferation. Yet, few innovations are ever truly unregulated and more often fall within the scope of one or more pre-existing regulatory regimes, a process referred to as ‘inherited regulation’. While the United Kingdom has enacted new legislation to specifically authorize and closely regulate MRTs, many jurisdictions will likely default to current oversight systems to manage advanced ARTs. This article evaluates and compares how several jurisdictions have already used four types of inherited regulatory regimes to manage MRTs and HHGE. Cases are drawn from jurisdictions where inherited regulatory interventions on advanced ARTs have taken place (USA, Greece, Ukraine, China, and Russia) and include jurisdictions closely connected with those cases (Mexico and Spain). When accounting for political, cultural, and religious contexts, many of these inherited regimes offer promise as starting points for governance of advanced ARTs, yet each will require further adjustments and tailoring to adequately manage the benefits and risks of these powerful innovations.

## I. INTRODUCTION

Technology has long influenced how and when human reproduction can occur, transforming and being transformed by everything from social values to economic structures in the process. Yet, over the past four-and-a-half decades, the actual or potential role of technology in reproduction has rapidly expanded.^1^ The birth of Louise Brown in 1978 through in vitro fertilization (IVF) catalyzed the clinical application of assisted reproductive technologies (ARTs) around the world^2^ as well as ethical and policy debates around their use.^3^ Incremental innovation in ART methods now occurs continuously, aiming to increase the success rates of pregnancy and live births through established fertility treatments. However, more radical or ‘seismic’ innovations in ARTs have also occurred since the turn of the millennium, making novel reproductive options possible with corresponding policy implications.^4^

These advanced ART techniques move beyond IVF and related methods by manipulating gametes, embryos, or even somatic cells at the organelle, genomic, or gene expression levels, ultimately producing a viable embryo biologically related to the intended parents. Advanced ART techniques began to arise over two decades ago with reproductive cloning and ooplasmic transfer, although the controversies they provoked resulted in legislative and regulatory actions to block their use in many jurisdictions.^5^ Unlike earlier advanced ARTs, which lack direct health benefits for the resulting children, newer advanced ARTs promise significant benefits to at least a small number of families who otherwise would be unable to have a healthy, biologically related child. Such techniques, including mitochondrial replacement therapies (MRTs) and heritable human genome editing (HHGE), have not yet achieved widespread use. Yet, the healthy births of multiple children from MRTs across a handful of jurisdictions and a single use of HHGE yielding three children in China foreshadows a coming time where the technologies may become more widely adopted.^6^

In this article, we trace how multiple states have already used available regulatory systems to steer the use of, or prohibit, certain advanced ART techniques. Scholars have already begun to make comparisons of several states’ approaches to one type of advanced technique, MRTs, addressing the matters of how jurisdictions conceptualize and categorize the technology or whether states have or will enact novel regulatory regimes.^7^ This article takes a different approach by comparing oversight responses to both MRTs and HHGE that are grounded in existing rules and regulatory authorities (rather than novel frameworks) and examining the type of regulatory regime applied in each case, assuming that the regulation of one form of advanced ART will undoubtedly inform decision-making around others. By focusing on how jurisdictions can use existing oversight mechanisms, conceptualized here through the lens of inherited regulation, we aim to illustrate how these advanced ARTs are not truly ‘unregulated’ and explore the normative dimensions of wielding inherited regulation in this space. As global debates continue over how states and the international community should respond to MRT and HHGE, comparative analysis and a recognition that advanced ARTs can trigger inherited regulatory regimes may offer insights relevant to policymakers and stakeholders more broadly.

In this article, we find evidence of jurisdictions taking multiple different approaches to using existing regulatory structures and instruments to manage advanced ARTs, and we argue that effectively using these regimes will require policymakers to thoughtfully, and incrementally, tailor their requirements to advanced ARTs. For some jurisdictions, this will require coordinating multiple inherited regimes, potentially at multiple levels of government. Section II provides an overview of two nascent advanced ARTs, which have already seen clinical use, before Section III introduces the theoretical framework of inherited regulation. Five case studies of inherited regulatory interventions on advanced ARTs are presented in Section IV, followed by a comparison and discussion of these cases in Section V. Concluding thoughts and implications for policy strategies around advanced ARTs and inherited regulation are set out in Section VI.

## II. OVERVIEW OF ADVANCED ARTS ALREADY IN USE

Two broad classes of advanced ARTs have recently seen clinical applications, with others currently under development. The first, MRTs, have gained prominence for their potential to prevent some mitochondrial diseases.^8^ Mitochondria produce energy for most human cell types, and mutations in the DNA carried by these organelles (mtDNA) can result in complex diseases that can affect several organ systems simultaneously, reducing the length and quality of life in more severe cases.^9^ Individuals inherit mitochondria from their biological mother through a ‘bottleneck’ effect, where egg cells obtain only a fraction of the mother’s total mitochondrial population.^10^ If enough dysfunctional mitochondria pass into an egg, the resulting child may have a mitochondrial disease even if the parents are asymptomatic.

**Figure 1:**
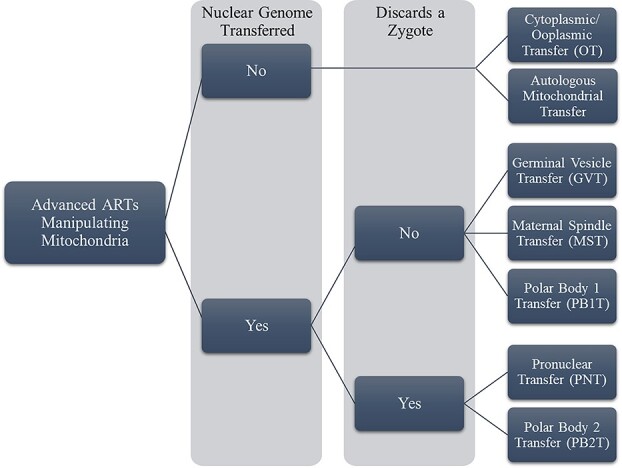
Advanced ARTs involving mitochondrial manipulation.

MRTs offer technologies to avoid such diseases by matching the mtDNA of a healthy donor woman with the nuclear DNA of the two intended parents, allowing parents to have a healthy, biologically related child. Prominent techniques include pronuclear transfer (PNT), maternal spindle transfer (MST), and polar body transfer (PBT), where PNT and one form of PBT require a fertilized egg to be discarded but MST and the other PBT variant do not.^11^Figure 1 reviews the broad categories of MRTs and other reproductive treatments involving mitochondrial manipulation.

Alongside their potential benefits, these MRT methods also present potential health risks and ethical concerns. Various mitochondrial-nuclear interactions occur in healthy cells, so pairing the intended parents’ nuclear genome with donor mitochondria could result in safety issues if potentially harmful mismatches occur.^12^ As MRTs often do not remove all parental mitochondria, various mechanisms during development could cause an embryo to revert to a state where dysfunctional mitochondria predominate.^13^ To date, these safety and effectiveness concerns have not been reported in the small number of children born through MRTs,^14^ although very little data on the health of these children have been peer-reviewed and published at this time. They do, however, remain as a hypothetical concern and, notably, at least female children born following MRTs could potentially pass the issues to their children. Several ethical, legal, and social concerns arise with MRTs as well, including the legal and social status of the donor as a parent or as an anonymous donor, whether to permit female children, and whether to empower non-heteronormative family structures (such as same-sex female couples) to use the techniques to have a child with genetic links to all parents.^15^ Further concerns include that developing MRTs will boost the social acceptability of nuclear germline editing.^16^ Additionally, some scientists and clinicians hypothesize these techniques could improve the success rate of traditional ARTs for some types of patients, such as women of advanced age. However, performing MRTs to treat infertility does not provide direct benefits to the resulting child^17^ and may alter benefit–risk calculations, especially as available evidence has shown little or limited improvement in ART success rates thus far.^18^

**Figure 2:**
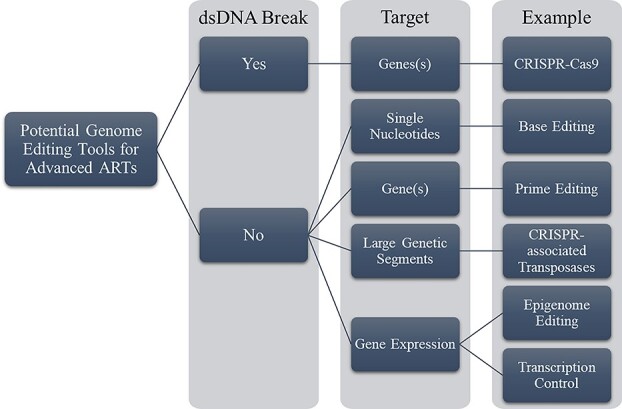
Genome-editing techniques with potential use in advanced ARTs.

A second advanced ART which has already seen clinical application is heritable human genome editing (HHGE).^19^ As opposed to MRTs, these techniques modify genetic material found within the nucleus of an embryo or gametes and so could alter the biological features of a resulting child beyond merely mitochondrial function. Editing the nuclear genetic material offers the possibility of avoiding a swath of heritable diseases as well as other questionably therapeutic interventions and overt nonmedical uses.^20^ The pre-eminent CRISPR-Cas9 platform operates by breaking both strands of DNA at a particular place in the genome and then relies on the cell’s genetic repair mechanisms to help remove, replace, or add DNA segments.^21^ However, cellular repair mechanisms may at times repair these double stranded DNA breaks in unintended ways, affecting the editing outcome. Accordingly, research on newer techniques that avoid double stranded DNA breaks, such as base editing and ‘prime editing’, is underway.^22^ An overview of these techniques with potential application in HHGE is presented in Figure 2.

Various stakeholders have raised multiple safety and effectiveness risks on the potential use of HHGE, including leading scientists Jennifer Doudna and Feng Zhang,^23^ particularly as edits would become heritable for subsequent generations. Both off-target and on-target mutations can occur, where the genome-editing tool alters genetic material other than the intended site or modifies the intended site in unexpected manners, respectively.^24^ Mosaicism presents another class of concern, where an embryo could come to have different sets of cells affected by the genome editing in divergent ways.^25^ Some mistakes may not lead to health issues, but more serious editing errors or complex mosaicism could lead to disease in the resulting child that would not have otherwise been present.

Myriad social and ethical concerns have also risen to meet the realistic potential of HHGE. These range from challenges over equitable access, human enhancement, and medical tourism to the rightful place of biomedicine in reproduction and the fundamental meaning of human dignity or self.^26^ Given these substantial concerns and notable health risks, multiple calls for a global moratorium on HHGE followed after its first use in 2018.^27^ A panel convened by the US National Academies and UK Royal Society concluded in 2020 that the most ethically acceptable use of the technology would prevent diseases with substantial morbidity and mortality, caused by defects in a single gene, where no other reproductive technology could prevent the disease in a biologically related child.^28^

Other advanced ARTs are being developed, though appear to be substantially farther from clinical application than MRTs and HHGE at this time. These might include potentially transformative innovations such as in vitro gametogenesis or artificial womb technology,^29^ though their distance from clinical application places them outside the immediate scope of this article.

## III. INHERITED REGULATION

Nascent technologies frequently incite legal, ethical, and policy challenges by posing novel benefits and risks—real or perceived—unanticipated by current systems of governance. The speed of innovation often appears to accelerate as a technology begins to emerge and its uses become more apparent, yet government entities create new legal norms at a relatively constant rate. The result is a ‘pacing problem’, where the governing activities of legislative and regulatory bodies increasingly lag behind technological innovation.^30^ However, this ‘legal lag’ in most cases will not necessarily result in a regulatory vacuum or substantive regulatory gaps^31^—despite often vocal and persistent claims to the contrary.

More often, existing regulatory regimes can, and do, apply to emerging technologies. The question is therefore about the efficacy of the current regime in achieving its stated policy goals and addressing emergent concerns rather than whether or not the technology and/or its products fall within the scope of existing regulation.^32^ Stokes describes this phenomenon as ‘inherited regulation’, illustrating the concept with a depiction of how European Union (EU) officials in the 2000s extended existing consumer products regulation to manage nanotechnologies.^33^ While EU consumer protection law lacked provisions that specifically referred to, or anticipated, nanotechnologies, the European Commission (EC) generally avoided new legislation because it felt current rules, institutions, and enforcement mechanisms provided the tools needed to oversee the entry of nanotechnology-based applications into its market.^34^ Even though the EU ultimately did modify their cosmetics regulation and food labeling rules to address certain types and applications of nanomaterials, these moves occurred as part of a broader regulatory reform agenda and largely tweaked existing regimes rather than establishing a ‘sui generis’ approach for nanomaterials.^35^ This approach of extending old regimes can apply to virtually any technological context, especially when the regulation targets broader outcomes such as safety or environmental protection.

Discourse on inherited regulation has focused on both the degree of coverage and the efficacy of past regimes for emerging technologies. Regarding coverage, inherited regimes may apply to new technologies depending on the scope of both the existing, written rules themselves, and on the interpretation of those standards. For example, an existing regime may cover the products of an emerging technology (ie product regulation), but not the novel processes involved in their production (ie process regulation), as is common in the USA.^36^ Coverage with inherited regulation can result in various types of rules being mapped onto the emerging technology, ranging from technical standards to full prohibition with criminal sanctions.^37^ Whether formal rules are written broadly or narrowly will help set the size of the net that inherited regulations can cast over new technologies.^38^ Further, how regulators and stakeholders interpret and apply those written rules—whether formally or informally—will also modify the capacity of inherited regulation to cover new technologies. Reinterpreting existing norms may be required for inherited regimes to apply, or apply more directly, which could involve formal procedures or less formal discussions or shifts in practice among regulators, regulatees, and stakeholders.^39^

When assessing an inherited regime’s fitness (ie efficacy) for an emerging technology, it becomes critical to interrogate the goals, assumptions, and structures underlying the existing regulatory system.^40^ Unquestioningly mapping such assumptions and values onto a new technology may result in a poor fit when stakeholders have prioritized other normative goals, such as risk management, above the objectives espoused by the inherited regime.^41^ The generally high uncertainty over the nature and extent of risks for emerging technologies may also contribute to a poorer fit when applying the tools of prior oversight schemes.

Evaluating fitness in regulatory inheritance therefore requires examining entire regimes, not just individual rules or standards. Levi-Faur defines a regulatory regime as the collection of all norms, values, assumptions, procedures, and actors engaged in regulation within a particular industry, sector, or space.^42^ Viewing full regimes can illuminate how assumptions and goals are passed on with regulatory inheritance better than merely examining written rules, as the context of existing rules will also translate to new technologies. For instance, from a functional perspective, emerging technologies should inherit not only specific standards but also the relevant regulatory institutions.^43^ Understanding that regulators, regulatees, and third-party stakeholders negotiate how principles and norms should apply in an inherited regime, driving fragmentation and complexity in the system, should further expand the scope of examination.^44^ Inevitably, the politics surrounding the inherited regime may clash with the politics surrounding the nascent technology.

Coverage and fitness can inform the extent to which inherited regulations will capture emerging technologies with appropriate goals and tools, however, evaluating the success of an inherited regulatory regime will also require looking to empirical evidence. Assessing regulatory interventions based on inherited regimes will therefore require analyzing the implementation of and compliance with the inherited oversight system. Implementation efforts will depend both on the quality of a regulator’s information gathering, as failing to detect the use of an emerging technology within their jurisdiction will prevent applying standards to its use, and on the enforcement strategy selected.^45^ Whether and how responsive the enforcement of inherited regimes are and the extent of communication with regulatees and third parties may alter regulatory outcomes as well.^46^ Neilsen and Parker explain how compliance with oversight systems depends on a superposition of social, economic, and normative motives, where the exact blend and constituent parts will vary with each regulatee.^47^ Critical for inherited regulatory regimes, however, is that compliance also depends on a regulatee’s knowledge of whether and how existing rules apply to new behaviors.^48^

Analyzing how well an inherited regulatory regime can govern an emerging technology therefore can be described as involving at least four components: coverage, fitness, implementation, and compliance. This article will proceed by using fairly established normative criteria—effectiveness and legitimacy—to assess and compare the performance of inherited regulatory interventions on advanced ARTs across five cases. Effectiveness of regulation measures, in relative terms, how well oversight activity achieves its policy goals while legitimacy describes the degree and character of social, moral, and political support behind the regulatory actions and institutions.^49^

## IV. CASES OF INHERITED REGULATORY INTERVENTIONS ON ADVANCED ARTS

Advanced ARTs pose numerous, complex policy challenges. Balancing health and social risks across generations against potentially powerful benefits to individuals, families, and health care systems calls for robust regulatory oversight. However, the regulatory inheritance discourse and lessons from other emerging technologies suggests that most jurisdictions will not establish new regimes specifically tailored to manage advanced ARTs.

Only one state has thus far constructed a novel regulatory regime for an advanced ART. In 2015, the United Kingdom became the first jurisdiction to enact legislation authorizing and setting out a new regulatory regime for MRTs.^50^ The change followed several years of debate, influenced by ethical, legal, economic, and medical inputs from both experts at the Nuffield Council on Bioethics, the Wellcome Trust, and public engagement, including with patient advocacy groups.^51^ Notably, the United Kingdom already had a robust ART regulatory system to build on, administered by the dedicated ART regulatory body for clinical and research uses of human embryos in Britain, the Human Fertilisation and Embryology Authority (HFEA).

The new MRT legislation empowered the HFEA to license qualified clinics seeking to provide MST or PNT techniques, solely to prevent mitochondrial diseases, where licensed clinics must further apply to regulators for approval to perform each treatment.^52^ The HFEA strongly encourages, but does not mandate, clinical follow-ups for children born through this advanced ART.^53^ The Newcastle Fertility Centre remains the only clinic to receive licensing and may perform only PNT under its license, although no known patients have yet undergone treatment at the Centre at the time of writing.^54^

**Figure 3:**
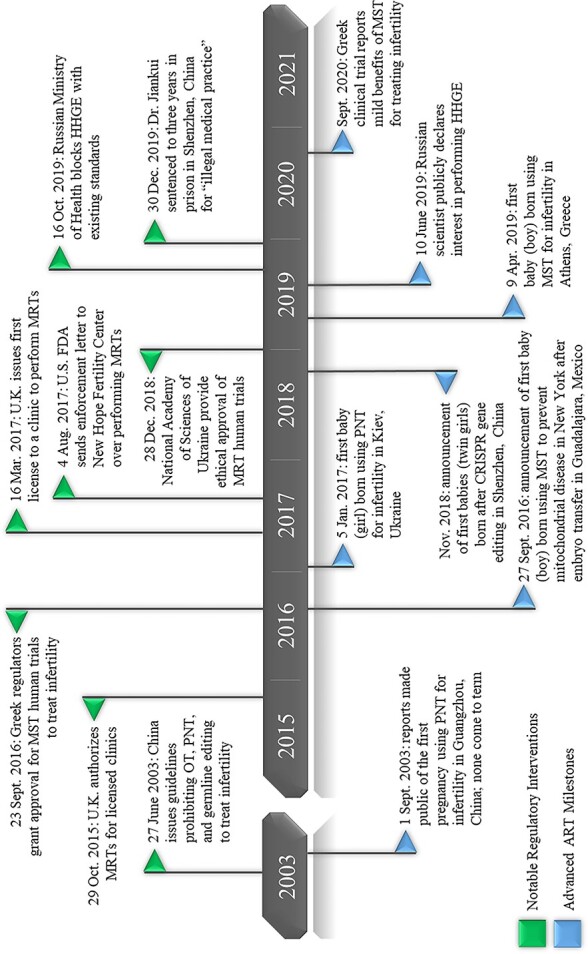
Timeline of advanced ART milestones and regulatory interventions.

While a handful of jurisdictions, including Australia and Singapore, are debating enacting similar regulatory regimes for MRTs,^55^ several others have already taken regulatory action on advanced ARTs without installing novel regimes. This section continues the conversation on inherited regulation by discussing the responses of these jurisdictions to advanced ARTs which have elected to apply existing oversight regimes. Cases are drawn from states where inherited regulatory interventions on advanced ARTs have taken place (USA, Greece, Ukraine, China, Russia) and include discussions of jurisdictions closely connected with those cases (Mexico, Spain). Figure 3 provides an outline of when significant reports of advanced ARTs appeared and when inherited regulatory interventions occurred.

### IV.A. USA and Mexico

In September 2016, news that the first baby had already been born following an MRT treatment sparked a global debate over the technology.^56^ The story involved a clinical team at the New Hope Fertility Center, led by New York-based Dr John Zhang, with sites in the USA and Mexico and a couple from Jordan, who had lost several pregnancies and two children to mitochondrial diseases.^57^ Later details established that the clinical team performed MST on the gametes, while in New York, before shipping the modified embryos to their branch in Guadalajara, Jalisco, for transfer to the Jordanian patient.^58^ The patient then returned to the New York clinic to give birth to a healthy baby boy.^59^ The clinic’s Institutional Review Board (IRB) in Mexico approved the MST method and informed consent protocol, which includes a follow-up plan through age 18; however, no IRB approval occurred in the USA for the MST performed in New York.^60^ The New Hope Fertility Center’s peer-reviewed article reported the child was healthy at 7 months of age, though little information has been released since then and the parents have declined to disclose information publicly or have their child’s mtDNA tested again ‘unless there is a clinical benefit’.^61^

Despite media attention in 2016 on a statement from a member of the clinical team that the procedure occurred in Mexico because the state has ‘no rules’ on MRTs, Palacios-González and Medina-Arellano point to relevant regulatory frameworks at both the federal and local levels.^62^ While Mexico lacks federal law or institutions which directly regulate clinical ARTs, including MRTs, the Regulations of the General Health Law on Health Research should still apply.^63^ Article 56 of these human subjects research regulations only permits clinical research on ARTs aiming to treat otherwise intractable infertility, subject to sanctions by the Federal Commission for Protection Against Sanitary Risk—Mexico’s regulatory body which broadly regulates the health industries.^64^ The existing regulatory framework could, therefore, potentially prohibit MRTs intended to prevent a mitochondrial disease but not MRTs intended only to treat infertility.^65^ At the local level, several states in Mexico protect human life from the moment of fertilization, including Jalisco, and at least some use criminal law to sanction violations.^66^ Notably, these criminal law regimes would only apply to MRTs which involve discarding a fertilized zygote (eg PNT and PB2T) and not others, including the MST used by the New Hope Fertility Center.^67^ Ultimately, however, Palacios-González and Medina-Arellano conclude that neither inherited regime could reach the New Hope Fertility Center’s conduct once it was discovered that the team performed the MST technique outside of Mexico,^68^ illustrating the difficulty of capturing transnational conduct with inherited regulation.

In 2017, the US Food and Drug Administration (FDA) took action by sending Dr Zhang a letter detailing the clinic’s violations of US medical products’ regulations.^69^ The team’s 2017 journal article indicated they had performed the MST technique while in New York City,^70^ placing those actions squarely within the FDA’s jurisdiction. Four months later, the FDA’s Center for Biologics Evaluation and Research (CBER) responded with the letter but did not detail specific sanctions for the violations. No further action has been taken by the FDA.

Currently, MRTs cannot lawfully be performed in the US, though the techniques are not specifically illegal. While the US National Academies recommended, in 2016, that clinical studies of MRTs to prevent mitochondrial diseases could proceed once evidence suggested adequate safety and efficacy, conditioned on using only male embryos initially, this plan has not yet moved forward.^71^ This barrier is a result of the US Congress having placed, starting in 2015, a rider in their appropriations bill funding the FDA which prohibits the agency from reviewing any application for ‘an exemption for investigational use . . . in research in which a human embryo is intentionally created or modified to include heritable genetic modification’.^72^ Since introducing a medical product to the market without FDA authorization would violate federal law on products regulation,^73^ the rider functionally prohibits MRTs.

The FDA has signaled it would oversee MRTs with its drug and biologics regulatory regimes should the appropriations rider be lifted, and FDA’s CBER cited drug and biologics rules when taking its enforcement action against the New Hope Fertility Center in 2017.^74^ The agency pursued enforcement action even though only the spindle transfer took place in the USA, reinforcing the FDA’s position that its regulatory target is the modified embryo itself.^75^ Both the drug and biologics regimes require product developers to go through premarket review with the FDA, starting by submitting an investigational new drug application before beginning clinical trials and ultimately applying for a biologics license application for MRTs.^76^ Further FDA guidance will likely be required on how the agency expects to interpret and apply these rules to MRTs, but the products regulatory regime has already been used to take enforcement actions on advanced ARTs.

### IV.B. Greece and Spain

Though not publicly known at the time, a clinic in Greece received state approval to move forward with MRT clinical trials only days before the announcement of the first MRT birth in New York.^77^ The Institute of Life clinic, in Athens, with the support of the Spanish firm Embryotools, proceeded with their clinical trial using the MST technique specifically to attempt to treat infertility rather than to prevent mitochondrial diseases. After patient recruiting began in 2018, the team reported its first successful birth (of a male infant) with the technique in April 2019.^78^ The Greek National Authority of Assisted Reproduction and a local hospital IRB provided approval for the clinical trial, which concluded in May 2020 with 25 total participants being involved.^79^ In October 2020, the clinical team reported they had achieved six pregnancies and at least five live births with MST.^80^ They concluded MST as a treatment for infertility may be effective but called for further studies to be conducted.

While most ART regulation in the EU occurs at the national level, some supranational legislation may apply to advanced ARTs such as MRTs. Most notably, Article 90 of the EU Clinical Trials Regulation, replacing the earlier Directive 2001/20/EC, prohibits clinical trials of medical products ‘which result in modifications to the subject’s germ line genetic identity’.^81^ Although this language could arguably apply to MRT programs, such as those in Greece and the United Kingdom (while it was subject to EU law),^82^ the EC quietly opined in late 2016 that MRTs do not involve ‘medicinal products’ and therefore would not trigger clinical trial laws.^83^ Though this EC opinion came in response to the United Kingdom’s 2015 legislation, the position would presumably apply to any move by Greek regulators as well. The Cell and Tissue Directive 2004/23/EC and its implementing legislation also provide safety and quality norms which generally apply to ARTs,^84^ though its standards do not specifically cover mitochondrial replacement or germline editing.^85^ Additionally, while Article 3(2)(b) the EU Charter of Fundamental Rights prohibits ‘eugenic practices’, significant uncertainty remains about whether this language applies to advanced ARTs.^86^

Further, Greece and Spain (but not the United Kingdom) have both ratified the Oviedo Convention, a multilateral agreement through the Council of Europe setting international norms on human rights in the biomedical context.^87^ Article 13 of the Convention prohibits ‘intervention[s] seeking to modify the human genome’ when they ‘introduce any modification in the genome of any descendants’. While this language readily applies to HHGE, whether the provision also captures MRTs depends on whether mtDNA is considered part of the human germline.^88^

Spain has an ART regulatory regime grounded in legislation from 2006, which establishes a licensing and registry system for ART clinics, basic standards for research and practice, and sanctions for noncompliance.^89^ The statute authorizes only four types of ARTs (artificial insemination, IVF, intracytoplasmic sperm injection, and gamete intrafallopian transfer), indicating that ARTs must pose limited risks to the mother and resulting child. To use any other form of ART, the National Commission on Human Assisted Reproduction, a national advisory body, must first issue a favorable report on the new technique, followed by regional or local health authorities authorizing its investigational use.^90^ The statute specifies that novel ARTs may only be approved through this process if they have a therapeutic purpose, with reasonable guarantees of treating or preventing the transmission of a disease, and do not modify ‘non-pathologic’, heritable elements of zygotes or embryos.^91^ The legislation appears to leave room for MRTs intended to prevent mitochondrial diseases, or treat infertility, if officials determine that sufficient evidence can demonstrate these ARTs will successfully achieve their therapeutic goals without substantial risk. HHGE to treat heritable diseases may also be permissible under the statute, should the National Commission and health officials approve it, though the statute appears to prohibit HHGE for nonmedical purposes. Given these more stringent and procedurally involved requirements, the Institute of Life and Embryotools team elected to pursue MRT treatment outside of Spain and suggested they would seek Spanish approval following successful clinical trials conducted elsewhere.^92^

Greece also has an existing regulatory regime for ARTs, overseen by the Greek National Authority of Assisted Reproduction, where ART treatments may only be performed to treat infertility or prevent serious heritable disease in a child.^93^ Established in 2005, the National Authority regulates ART clinics in Greece through a licensing program, including a national registry of licensed clinics.^94^ Clinics must submit performance data to the National Authority, which reviews licensed clinics every 3 years and has the power to sanction noncompliance with its standards, including through revoking a license. Further, proposed research resulting in a human pregnancy and research on human gametes or embryos requires approval from the National Authority before it may proceed.^95^ The legislative framework also places criminal and administrative sanctions on reproductive cloning, sex selection unless performed to avoid a heritable disease, and modifying the genome of human gametes or embryos.^96^ These provisions would likely prohibit HHGE, and perhaps even research involving genome editing of human embryos, but could accommodate MRTs for either avoiding disease or for treating infertility if mtDNA is not considered part of the human genome.

The existing Greek ART regulatory regime appears to grant the National Authority the powers necessary to approve a clinical trial for MRTs to treat infertility (and likely for the intended use of avoiding disease as well). The legislative provisions requiring regulatory approval before research leading to a pregnancy and mandate that ART interventions should treat infertility or prevent a heritable disease, alongside the absence of a direct prohibition on techniques involved in MRTs, should enable the approval of investigational MRTs at licensed ART clinics in Greece. While the Hellenic National Bioethics Commission recommended against clinical trials of MRTs in 2017,^97^ the National Authority still appears to retain the legal discretion to enable such trials. Moreover, compared to Spain’s approach of prohibiting ART techniques unless expressly authorized, Greece has a relatively permissive ART regime valuing reproductive autonomy.^98^ The current Greek regulatory framework had already been enacted before the 2016 decision by regulators to allow the clinical trial with the Institute of Life clinic and Embryotools to proceed.^99^ Therefore, the National Authority appears to have applied instruments available under its existing regime to oversee this advanced ART in Greece and is likely empowered to authorize further clinical trials for MRTs either to avoid disease or treat infertility.

### IV.C. Ukraine

The second known birth from an MRT occurred in January 2017 at the Nadiya Clinic in Kiev, Ukraine.^100^ Led by Dr Valery Zukin, the clinic performed PNT for a patient with intractable infertility, leading to the birth of a female infant. The Nadiya Clinic reported receiving ethical approval to perform PNT from the Ukrainian Association of Reproductive Medicine, a professional society.^101^ At least eight other children have since been born through MRTs through the Nadiya Clinic, with several other pregnancies reported.^102^ Following this first birth, the clinic joined with Dr John Zhang’s team in New York to form Darwin Life-Nadiya and offer PNT services to patients both from Ukraine and abroad for a fee of approximately US$8,000 or US$15,000, respectively.^103^ At least one foreign patient, from Sweden, has received treatment from Darwin Life-Nadiya resulting in a pregnancy.^104^ However, preliminary data from the Darwin Life-Nadiya trial in 2019 showed MRTs provided no benefit to fertility success rates in patients of advanced maternal age, and their report recommended not offering MRTs to this patient group to treat infertility.^105^

While the exact regulatory status of the clinical research is murky, the Nadiya Clinic claims to have received authorization to conduct a clinical trial from the Ministry of Health of Ukraine via a local hospital.^106^ A clinical trial registry appeared online in 2019 describing a 5-year trial for MRTs to either treat infertility or prevent mitochondrial disease, with patient recruitment ongoing and ethical review provided by the Commission on Bioethics of the National Academy of Sciences of Ukraine in December 2018.^107^ The registry also describes a protocol to have a pediatrician follow-up with children born through the study every year until the age of 7 and then once every 2 years until the age of 18, although it does not specify what follow-up will entail. As of August 2021, the clinical team has not yet published peer-reviewed reports from the clinical trial.

While Ukraine does not currently have comprehensive ART legislation,^108^ the Ukrainian Civil Code grants individuals the right to undergo ART treatment for medical purposes and Article 48 of Ukraine’s central health statute provides that ART techniques must be performed in compliance with standards issued by the Ministry of Health.^109^ The most recent set of ART technical standards was issued by the Ministry of Healthy in 2013, although they do not directly reference MRTs, HHGE, or germline modification generally.^110^ While the standards permit ART clinics to use donated oocytes to prevent a heritable disease or treat intractable infertility, they do not indicate whether these oocytes may be used as mitochondrial donors. Clinics offering ARTs must be licensed by the Ministry of Health to provide medical services, then accredited by the Ministry after 2 years of operation, and have a list of equipment and personnel prescribed in the ART technical standards.^111^ At minimum, these licensing requirements and technical standards should apply to the Nadiya Clinic’s use of MRTs, granting the Ministry of Health a degree of control over the clinic’s behavior.^112^

Other ART-related law in Ukraine potentially applicable to advanced ARTs do not appear to capture MRTs. Though Ukrainian legislation specifically forbids human reproductive cloning, the language is likely too narrow to apply to other forms of advanced ARTs.^113^ In 2018, draft national legislation was introduced, which would create a more formal ART regulatory framework and explicitly authorize MRTs and ooplasmic transfer, but failed to gain support.^114^ Moreover, Ukraine has signed but not ratified the Oviedo Convention,^115^ though its applicability to MRTs remains uncertain.

As the Darwin Life-Nadiya group claims to have received approval from the Ministry of Health to conduct a clinical trial on MRTs, Ukrainian health research regulations should also apply. Generally, Ukrainian health legislation provides basic requirements for medical research such as informed consent.^116^ Clinical trial regulations in Ukraine do require research protocols to be approved by both the Ministry of Health and a local ethics committee,^117^ but they appear to apply primarily to medical products and may not capture investigational ARTs in their scope.^118^ While ‘the Ministry of Health does not currently have a central body performing ethics review’ for ART trials, the Ministry ‘interacts with local bioethical committees’ at medical and research institutions to provide ethical review.^119^ This might explain why the Darwin Life-Nadiya group states they have obtained ‘permission’ from the Ministry of Health through a local hospital.

Furthermore, the National Academy of Sciences of Ukraine appears to have taken a rare step by providing ethical review for the Darwin Life-Nadyia group’s MRT clinical trial, although ethical approval came nearly 2 years after the first birth in Ukraine was reported.^120^ Their Commission on Bioethics ‘performs ethics review as an exception and only in highly specific areas of medical disciplines’ and provides ‘independent review’ separate from the Ministry of Health or local ethics committees.^121^ In response to a request for information filed by the authors, a spokesperson for the Commission on Bioethics indicated:

When making the decision, our Committee resolved that, according to all international acts, every person has the right to reproduction. The main setback for development of reproductive nuclear transfer techniques is the uncertainty of impact of this technology on a future child’s health. Considering it, we are not able to answer this question without conducting the experimental study on condition of patients’ consent and their understanding of possible risks.^122^

The rationale provided suggests interests in reproductive autonomy were judged to outweigh safety concerns for consenting patients in approving the clinical trial, particularly in light of the uncertainty around safety risks.

### IV.D. China

In 2003, a scientific conference abstract unexpectedly reported that the first pregnancy derived from PNT had been successfully established for a woman in China.^123^ The team, again involving Dr John Zhang, had performed the MRT to treat infertility, but none of the three fetuses came to term.^124^ The Chinese Ministry of Health swiftly responded to these reports with new clinical guidelines prohibiting nuclear transfer as well as ooplasmic transfer.^125^ These guidelines therefore intentionally cover at least PNT, and likely other MRTs as well, though only when the techniques are performed to treat infertility.^126^ The Ministry’s guidelines also specifically prohibit gene editing to treat infertility.^127^ However, Ishii notes the standards, legally classified as ministerial guidelines, as less directly enforceable than codified legislation or regulation.^128^

The National Health Commission (NHC), the current successor to the Ministry of Health, functions as the national ART regulator for China and all ART clinics must undergo certification and monitoring by the agency to offer services.^129^ The NHC holds at least some power to enforce its ART ministerial guidelines, including the 2003 standards on embryo modification, most significantly through revoking the license of a clinic found in violation.^130^ However, the implementation of this ART regulatory framework, as well as human subjects regulation, has been fragmented between various national bodies and local officials with limited coordination.^131^ In practice, the NHC’s enforcement activities do not appear to have kept pace with the expansion of ART clinics and services offered in China, and some prohibited services remain available.^132^ Separate from the ART regime, individual clinicians are also required to register and obtain proper licensing from the NHC, which has the power to deny and revoke licenses.^133^

In late November 2018, Antonio Regalado at the MIT Technology Review broke news that a group in China had performed HHGE with CRISPR-Cas9 tools, leading to the birth of twins and (later discovered) at least one other child.^134^ Dr He Jiankui and colleagues edited the *CCR5* gene in embryos from couples where the biological father was HIV-positive, given the gene’s role in HIV transmission, allegedly to decrease the risk of infection to the children throughout life.^135^ The unexpected, sudden revelation incited global criticism for violations of multiple scientific and medical norms.^136^ These included unacceptable risk for too little benefit, inadequate ethical review and consent protocols, potentially predatory patient recruitment, opacity, and failure to engage with the public, national officials, or international community before proceeding.^137^ Limited data have been released on the three children or their current health, although multiple concerns have been raised over potential health risks or complications in the near- and far-term.^138^ A scientific report was never published, as multiple journals rejected the submission, though some excerpts have become available.^139^

In performing HHGE, Dr He and his team appear to have violated not only the 2003 guidelines on embryo modification but also several other guidelines on ethical review and biotechnological research safety.^140^ While the NHC could have enforced its ministerial guidelines by sanctioning the clinic, most reporting on the incident suggests that the doctors and clinics involved in the embryo transfer procedures were unaware the embryos had been gene edited.^141^ Further, revoking a clinic’s license would not directly sanction He’s team or their conduct. While not directly under the ministerial guidelines, the NHC did take steps against He and colleagues by prohibiting them from performing ARTs in the future,^142^ likely through its licensing power. Additionally, the Ministry of Science and Technology forbid the three from obtaining public funding for future research.

The Chinese regulatory response to this case of advanced ARTs also relied on criminal law; in part, in response to political pressure from the international community to take definitive action against He.^143^ On December 30, 2019, a state media agency released news of the conviction and sentencing of He and his two colleagues after prosecution by authorities in the Guangdong Province.^144^ The court fined He 3 million yuan (about US$430,000 at the time) and sentenced him to 3 years in prison, with the colleagues receiving lighter sanctions. While the news release noted that the three had violated national ‘regulations and ethical principles’, presumably referring to the 2003 ministerial guidelines among others, the three were tried for ‘illegal medical practice’.^145^ Codified as Article 336 of the Chinese criminal code, the statute prohibits providing medical services ‘without obtaining the qualification for medical practice’ and can result in a sentence of up to 10 years in prison if ‘serious harm’ results.^146^ Lei and Qui suggest that officials used this charge because it was the only criminal provision available, as violations of the NHC guidelines cannot be directly enforced with criminal law.^147^ Further, they note that the court’s reference to the guidelines may have been an attempt at extending the application of the criminal code to this new situation. While the closed-door trial appears to have provided appropriate procedural protections to defendants, no judicial opinion with a legal rationale has been publicly released.^148^

In response to the 2018 incident, China issued new regulations on HHGE, in mid-2019, which would be more directly enforceable than ministerial guidance.^149^ In December 2020, a new subsection was added to Article 336 of the criminal code to punish ‘[implanting] any genetically edited or cloned human embryo into the body of a human being or animal’ with sentencing of up to 7 years in prison possible ‘if the circumstances are serious’.^150^

### IV.E. Russia

Only months after the He Jiankui incident, a scientist from Russia announced that he also intended to transfer gene-edited embryos to patients. In a statement to ‘Nature’ published in June 2019, Dr Denis Rebrikov revealed a strong intent to perform HHGE with a similar plan to Dr He’s, again targeting the *CCR5* gene with CRISPR tools.^151^ Here, however, clinicians would instead use the technique for couples where the biological mother was HIV-positive and nonresponsive to HIV medication. Rebrikov defended the proposed intervention as more ethical than Dr He’s protocol because it offered the potential benefit of reducing the risk of HIV transmission to the fetus during gestation, though commentators still raised strong concerns about overwhelming safety risks.^152^ By July 2019, Rebrikov’s main interests had shifted to using CRISPR tools to prevent heritable deafness, arguing this would address a medical necessity, and was in contact with at least one couple who could benefit from such an intervention.^153^ Rebrikov indicated several times that he would seek approval from the Russian government for both proposed HHGE interventions, though intimated at least once he might proceed beforehand if safety risks could be mitigated.^154^

While Russia does not have a dedicated ART regime, its general health statute creates a right to ART treatment and broadly empowers the government to approve and restrict ART protocols.^155^ Further, ART clinics must obtain a license from the federal government to operate and provide services.^156^ In 2012, Russia’s Ministry of Health issued an order detailing the approved ART services and protocols fertility clinics could provide, restrictions on those services, and requirements for clinics and laboratories.^157^ While this order carries detailed, technical prescriptions for authorized ART practices, neither the order nor the general legislation specifically address HHGE.^158^ Other Russian legislation, including on genetic engineering generally and on reproductive cloning, also does not address nuclear genome editing as an ART.^159^

In October 2019, responding to international pressures and in the shadow of the He Jiankui incident, the Russian Ministry of Health extended its 2012 order on ART protocols to preclude the clinical use of HHGE.^160^ To reach this decision, the Ministry appears to have interpreted the absence of a reference to clinical germline editing in the order’s detailed prescriptions as precluding their ability to approve HHGE. However, this indirect method of preventing HHGE does not specifically render the practice unlawful and the Ministry has acknowledged a need for more comprehensive oversight.^161^ In announcing its decision and reinterpretation of the ART regulation, the Ministry indicated its strong support of the World Health Organization (WHO) position that HHGE would be ‘premature’ at this time.^162^

In the wake of the Ministry’s decision, Rebrikov expressed disappointment but signaled he would not proceed without regulatory approval.^163^ Instead, he would continue with laboratory work to collect more data on the safety, effectiveness, and reliability of his proposed germline modifications, with the expectation that approval would quickly follow if data demonstrated safety.^164^

## V. COMPARISON AND DISCUSSION

These five cases of regulatory interventions on advanced ARTs with inherited regimes exemplify how jurisdictions can extend existing rules to emerging technologies. However, the legal question of whether current regimes can be extended is different from the normative inquiry into how well inherited regulation operates—and with what consequences. Previous literature analyzing inherited regulation has focused on nanotechnologies, finding that several jurisdictions successfully applied or extended their available regulatory regimes but with questionable fitness for managing the novel products.^165^ By adding comparative and empirical elements, this study aims to extend those analyses by drawing out lessons for advanced ART policymaking with potentially cross-jurisdictional applicability while seeking out more generalizable insights for the governance of emerging reproductive technologies and theory on inherited regulation.^166^

This section will assess the five case studies presented above (and reviewed in Table 1) using the analytical criteria for regulatory inheritance discussed in Section III: (i) coverage, (ii) fitness, and (iii) implementation and compliance. Of course, comparative legal and regulatory analysis is inevitably limited in the presence of an incomplete understanding of the broader sociocultural and historical context of each jurisdiction’s system.^167^ Differences in public opinions on advanced ARTs within, and between, jurisdictions can also vary widely and continuously shift, introducing further complexity.^168^ Accordingly, this discussion will aim to proceed lightly and rely on observations of how each system has been applied on the ground where possible.

**Table 1:** Comparison of Inherited Regulatory Interventions on Advanced ARTs

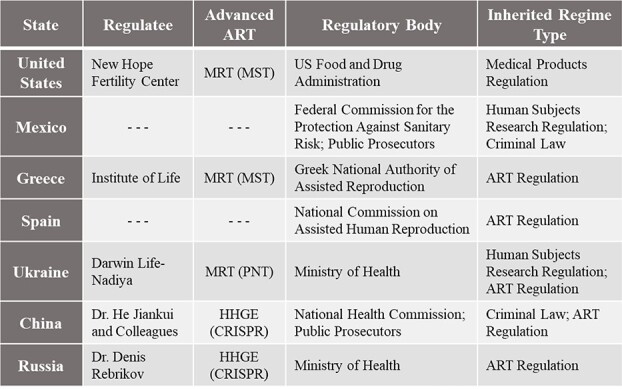

### V.A. Coverage

The effectiveness of inherited regulation begins with coverage, the legal and political processes of extending previous rules and regimes to emerging technologies through (re)interpreting current regulatory norms. The degree to which an inherited regime can cover new technological spaces will depend on the scope and targets of existing rules and political support for formally or informally applying those rules instead of crafting new ones.^169^ The seven jurisdictions in the case studies above could or have achieved coverage of advanced ARTs through extending four types of regulatory regimes.

First, the most common type of inherited regime applied to emerging reproductive technologies were existing ART regulatory systems. Extending these systems reflected a pragmatic choice by policymakers, as the scope of these regimes often readily captured the new techniques. However, the differences in the breadth and scope of the ART regimes in each case required some jurisdictions to perform more interpretive work than others in defaulting to these regimes. For example, Spain’s system of national and local approval for any new reproductive technique and Greece’s national regulatory approval before clinics conduct human research represented the most robust ART regimes, readily capturing MRTs.^170^ China also readily applied its system by forbidding members of the HHGE team from performing ART in the future, although the researchers were never health care workers.^171^ Rather than comprehensive legislation, Russia and Ukraine each had a narrower set of technical ART standards. Russia successfully reinterpreted its technical standards to prohibit HHGE, while Ukraine made no clear effort to do so for MRTs,^172^ illustrating the need for political appetite—not merely legal possibility—to achieve coverage with an inherited system.

Second, two jurisdictions did or could use criminal law to oversee advanced ART activity: China and Mexico.^173^ By prosecuting Dr He and two associates for ‘illegal medical practice’, officials in China at the provincial level, through the courts, were able to impose more targeted and severe sanctions than those available under ART rules.^174^ This example illustrates the potential utility of engaging multiple existing regimes to achieve more comprehensive coverage in the oversight of an emerging technology space, particularly when individual regulatory regimes might have structural limitations on the types of targets or regulatees subject to rules. In Mexico, some subnational governments have criminalized destroying embryos in ways that would almost certainly extend to some MRTs as well, though different standards would apply in different Mexican states.^175^ The use of criminal law and public prosecutors at the local level in China and Mexico highlight how subnational governments and judicial systems, not merely national governments and regulatory agencies, can not only play a role in achieving coverage in regulatory inheritance but could also drive fragmentation within a national jurisdiction.

Third, Mexico and Ukraine achieved different extents of coverage for advanced ARTs with human subjects research regulation, although these regimes can only address research and not clinical/commercial uses of the techniques. Mexico’s rules should prohibit the study of any new ART technique unless it treats infertility, which should readily cover MRTs intended to prevent disease and HHGE and benefit from a national regulatory agency to enforce them.^176^ By comparison, Ukraine has less-comprehensive human subjects research regulations which appear to require little more than informed consent and local ethical board approval for clinical ART investigations. The National Academy of Sciences of Ukraine bolstered this coverage, albeit in only a quasi-formal fashion.^177^ Further, health research oversight by definition excludes clinical practice from its scope, targeting researchers instead of clinics or markets. The ‘clinical trial’ in Ukraine blurs the lines between research and clinical practice by actively marketing MRT services for a fee,^178^ illustrating the structural limitations in coverage offered by this type of system.

Finally, only the USA elected to extend products regulation to advanced ARTs, with the FDA enforcing drug and biologics rules on MRTs and signaling those norms would also apply to HHGE.^179^ The use of a products regulatory regime marks a departure from other jurisdictions, such as the United Kingdom and EU which have both claimed that MRTs do not constitute products.^180^ Applying a products regime to an advanced ART required interpreting and extending regulatory norms on medical products to apply to embryos, although the FDA had already extended products norms to embryos in reproductive cloning and ooplasmic transfer two decades ago.

More generally, coverage in several cases struggled to cope with medical tourism, as national or subnational regimes could not easily apply transnationally.^181^ The Darwin Life-Nadyia clinic in Ukraine has provided MRT services to patients from jurisdictions where this treatment would likely be prohibited or simply not available.^182^ Similarly, the Institute of Life appears to have selected a European jurisdiction with inherited rules and political conditions more amenable to their research agenda (Greece) over another state where part of the team conducted work regularly (Spain).^183^ The USA–Mexico case provided an example of one state extending existing rules to cover advanced ARTs when the other state’s inherited standards could not attach due to jurisdictional constraints. While this may have occurred without deliberate coordination between the USA and Mexico, jurisdictions cooperating to apply national inherited regulations in concert may provide at least one path forward in addressing medical tourism. This may prove to be effective even if each state applies a different type of inherited regime to achieve coverage, as occurred with the USA (products regulation) and Mexico (human subjects research regulation). Whether and how jurisdictions coordinate their inherited regulatory programs offers research questions meriting future inquiry.

Pertaining to the theory of inherited regulation, several cases demonstrate that extending existing regulatory regimes to an emerging technology is not merely a passive process or an inevitable result. Rather, an emerging technology will only inherit a prior regime through actors in the regulatory community actively (re)interpreting available regulatory norms to apply in unforeseen settings, constituting an inherently political and contestable process.^184^ For example, the Greek National Authority took action to approve an MRT clinical trial to treat infertility using its existing rules, in 2016, despite the knowledge that the UK legislation enacted the year prior was contested by some British and international stakeholders.^185^ Greek regulators did not change their course even after increased public scrutiny on MRTs, following the birth of the first child at the New Hope Fertility Center, or after the Hellenic National Bioethics Commission published a report the following year advising against clinical trials.^186^ The Russian Ministry of Health determined its current rules would cover HHGE only after an extend process with political pressures from stakeholders both domestically and in the international community.^187^ Moreover, the Ministry achieved this result only after interpreting the lack of a reference to HHGE in available standards as not permitting the techniques, which commentators noted could be perceived as flimsy legal reasoning.^188^ These cases illustrate how both the advent and degree of coverage by an inherited regime depends on the active efforts of regulators and stakeholders, where successful coverage could be derailed or slowed by conflicting interests and values. Just as the ‘pacing problem’ is not inevitable in the governance of emerging technologies,^189^ neither is the extension of existing oversight regimes.

### V.B. Fitness

The case studies saw four different types of inherited regulatory regimes applied to achieve coverage of advanced ARTs. However, the degree of coverage achieved and the appropriateness of an existing regime are two separate and equally important questions. Determining the fitness of an inherited regime requires normative inquiry into how appropriate its assumptions, values, and goals are for managing the benefits and (potential) risks—including short- and long-term—associated with an emerging technology.^190^ Each type of existing regulatory regime, when contextualized in the social, cultural, and political histories and present of a specific jurisdiction, will inevitably present advantages and disadvantages when deployed in response to a new product or process. Identifying these normative strengths and weaknesses will assist decision-makers in tailoring an existing regulatory regime to effectively capture an emerging technology.^191^ Or, where fit is determined to be poor, policymakers can use such a determination as the basis for developing a new, more tailored regulatory response.

Five jurisdictions extended ART regulation, which generally seeks to promote the safety of patients and children, effectiveness of treatments, and certain ethical principles or social values such as reproductive autonomy.^192^ Legislation in Spain and China’s ministerial guidelines specifically list goals of safety and effectiveness for families and future generations, with Greece taking a similar approach but emphasizing autonomy.^193^ Russian and Ukrainian legislation providing individual rights to access infertility treatment in tandem with empowering governments to issue standards on or approve ART techniques also appear to reflect these goals.^194^ Secondary goals often include encouraging responsibility in ART research^195^ for which the Spanish, Greek, and Chinese systems explicitly provide oversight. While stakeholders may contest how jurisdictions implement and extend ART regulation, the most robust of these systems offer high relative fitness for inheritance by emerging reproductive technologies. The broad scope from research through clinical practice, assumption that ethical norms matter in addition to safety and effectiveness standards, and goals of risk management with technical standards make it possible for existing regimes to not only prohibit undesirable advanced ARTs but also to guide more responsible development and uses of permitted techniques.

Human research subjects and ART regulatory regimes both seek to promote safe and ethical uses of ARTs, yet their different assumptions reduce the fitness of the former. Broadly, human subjects regimes aim to ensure that research is conducted consistent with applicable bioethical principles and that the rights of participants or communities are protected.^196^ Common bioethical principles, such as beneficence and nonmaleficence, can promote behaviors similar to those sought by the safety goals of ART regulation.^197^ However, human subjects oversight generally makes the assumption that commitment to bioethical principles will promote safety outcomes without more prescriptive technical standards. The determination in Ukraine that MRT trials could move forward merely after obtaining informed consent,^198^ without further specifications such as for medical follow-up with children, illustrates how broad bioethical principles can be interpreted with variable results and may yield oversight perceived as shallow. Especially when techniques pose highly similar ethical concerns, such as two forms of MRTs that do not require discarding an embryo, human subjects regulation may struggle to achieve nuanced oversight. For example, human subjects rules in Mexico likely block advanced ARTs not intended to treat infertility but provide no tools to guide the development and clinical use of MRTs used as infertility treatments.^199^

Only the USA defaulted to a pre-existing system of medical products regulation for advanced ARTs, although it is notable that the USA lacks a national system of ART oversight. Medical products regulation shares both safety and effectiveness goals with ART regulation but makes different assumptions with impacts on fitness. Though both use technical rulemaking and enforcement, products regimes typically assume that products will indeed enter the market once safety and performance thresholds have been met.^200^ For example, no US federal law explicitly prohibits human reproductive cloning, and the USA has instead applied existing products regulation through the FDA to manage this advanced ART.^201^ Although FDA management of cloning does create a ‘de facto’ prohibition, this approach does not formally prohibit cloning and, at least hypothetically, provides the infrastructure for approving the technique in the future. Applying products regulation to other advanced ARTs would similarly propagate the assumption that threshold safety and effectiveness are the only norms relevant to approving new techniques without direct consideration of ethical or social issues.

China and Mexico both had criminal law applicable to advanced ARTs, though we argue that criminal law offers poor fitness for the oversight of these technologies. Criminal law generally examines behavior through a moral lens, with common rationales such as deterrence of or retribution for acts perceived as immoral.^202^ For example, the Chinese criminal code specifies the body of law strives to ‘punish crimes and protect the people’, property, and social welfare.^203^ This moral motive stands in contrast with the more instrumentalist goals common in other regulatory regimes seeking effective achievement of predetermined objectives, such as risk management.^204^ While criminal law clearly has a regulatory function, the assumption that enforcement should only occur in the context of an immoral act could lead to blunt or uneven management of complex advanced ARTs. The prosecution of Dr He and colleagues in China for practicing medicine without a license ultimately only establishes a norm that non-physicians may not perform advanced ARTs, leaving uncertainty for if or how physicians could appropriately do so.^205^ Similarly, subnational laws in Mexico criminalizing destroying an embryo may block certain MRT methods on moral grounds but offers no mechanism to steer the responsible use of other advanced ARTs.^206^

More broadly for inquiry on inherited regulation, these cases also demonstrate that jurisdictions may at times have multiple pre-existing regimes from which to select when managing an emerging technology, and each regime type may have different fitness considerations. Mexico, Ukraine, and China each had two types of regulatory regimes applicable to advanced ARTs, and China took enforcement measures through both its ART regulatory framework and its criminal legal system.^207^ Different inherited regimes available in the same jurisdiction may offer divergent regulatory instruments to achieve their goals or be located at different levels of government, potentially impacting fitness assessments. The cases here illustrate a variety of regulatory tools made available through different regime types, including technical standards for ART techniques or medical products, ethics review, regulatory approval before beginning clinical trials, and full prohibition backed by civil or criminal sanctions.^208^ Assuming similar levels of coverage are possible with multiple inherited regime types, jurisdictions will need to make decisions about which regulatory frameworks (or combinations thereof) to extend to emerging technologies. This conclusion suggests that scholars and policymakers interested in inherited regulation should aim to not only analyze the fitness of any one inherited regime in a given jurisdiction but also to identify other potentially applicable regime types and compare their normative goals and technical instruments.

Further, determining whether and how to activate potentially overlapping inherited regimes will inevitably introduce coordination problems into the use of inherited regulation, both within and across different levels of government.^209^ The case of HHGE in China involved both national-level ART regulators and provincial-level prosecutors taking enforcement actions against the scientists who performed the technique under different legal frameworks, while Ukraine had two national-level regulatory frameworks applicable to MRTs. These observations raise new questions for the theory of inherited regulation around how to best coordinate multiple inherited regimes in a single jurisdiction, whether decision-makers intentionally or unintentionally deploy multiple regimes, and how to resolve conflicts between regulatory bodies with divergent mandates and types of expertise or between national and subnational bodies applying inherited regulatory regimes. Future theoretical and empirical research will be required to analyze these orchestration issues in inherited regulation and to consider how to develop productive policy mixes of inherited regulatory regimes.^210^

### V.C. Implementation and Compliance

The effectiveness and legitimacy of inherited regulatory regimes depend not only on their coverage and fitness for the new technology (or product) but also on how policymakers implement the inherited system and how regulated actors respond. Factors, such as whether and how regulators communicate substantive expectations to regulatees and monitor conduct, procedural actions taken in enforcement, and political environment, may all modulate policy outcomes when using an inherited regime.^211^ How regulatees behave and comply with inherited regimes will also affect outcomes. This begins with whether regulatees realize that inherited norms could apply to their activities and, if so, that compliance behaviors will likely be mediated by their economic, social, and normative motivations.^212^ Ultimately, measuring the effectiveness and legitimacy of inherited regulation will depend on policy objectives and sociocultural context, but some general observations can be drawn from the cases of advanced ARTs in this study regarding regulator-regulatee communication, the role of politics, and enforcement style.

First, one trend observed in the cases was that early, honest communication between regulators and regulated entities on how inherited regimes would apply to advanced ARTs led to more positive outcomes. For example, regulatees in the Greece and Russia cases both took affirmative measures to contact regulators before proceeding with advanced ARTs, signaling their knowledge that existing requirements could apply and some level of respect for the relevant regulatory authorities. Communicating with officials in advance afforded regulators an opportunity to consider whether and how to extend existing rules to cover the advanced ART, avoiding snap decisions and regulatory outcomes perceived as unpredictable. The Institute of Life’s MRT activities appear to have remained within the scope of what Greek regulators approved, illustrating an effective inherited regulatory intervention which still allowed an advanced ART to proceed. Dr Rebrikov in Russia expressed disappointment at the Ministry of Health’s decision to block HHGE but did appear to accept the outcome as being legitimate.^213^ The Darwin Life-Nadyia clinic also appeared to seek input from the National Academy of Sciences of Ukraine when offering MRTs,^214^ although the regulatory response was less comprehensive by comparison and few documents or details have been made publicly available.

By contrast, the regulatees in China appeared to be unaware that inherited regimes could cover their use of HHGE. The team’s proud announcement of their achievement with the advanced ART, to the apparent surprise of health officials, suggests the team failed to communicate with the NHC or other officials about their plans.^215^ Further, reports of the incident depict Dr He as believing officials would celebrate their success with HHGE.^216^ While the subsequent use of criminal law achieved greater inherited regulatory coverage of the advanced ART, criminal prosecution was clearly not the response expected by Dr He and colleagues. Policymakers signaling in advance how they might apply inherited rules, and who they might apply to, as well as regulated entities communicating with regulators before deploying emerging technologies could help avoid outcomes that could be perceived as unpredictable. Open, early communication between regulators and the advanced ART community will only become more important as patient interest and market demand for these services increase.

However, willful noncompliance and insufficient monitoring were still possible after regulators communicated their substantive expectations to the public at large. The US FDA publicly stated in early 2016 it would not approve MRTs after US lawmakers had removed their budget to do so, setting a clear expectation for ART researchers and clinicians within the jurisdiction.^217^ Later, in 2016, after already performing MRTs once without regulatory approval, the New Hope Fertility Center contacted the US FDA about authorization for clinical trials.^218^ Despite the team indicating it would not perform MRT techniques in the USA until receiving FDA authorization, the clinic continued to advertise its MRT services, precipitating the FDA’s enforcement action.^219^ This recalcitrant behavior appeared to be part of a pattern, as the clinical team also failed to meaningfully engage with health officials in Mexico prior to providing part of the MRT treatment there.^220^ This example illustrates how effectively implementing inherited regulation still requires monitoring and enforcement activities, not merely standard setting, to achieve effective coverage of a new technology.

Second, decisions around how to implement inherited regulatory interventions for advanced ARTs occurred against the backdrop of domestic and global political tides. China received intense criticism from the international scientific and political communities after HHGE was performed within its jurisdiction, and Hurlbut argues that China applied harsh sanctions from criminal law to appease external actors and restore its reputation as much as to achieve internal policy goals.^221^ Similar forces placed pressure on Russia to prohibit HHGE under its existing rules when Dr Rebrikov announced his interest in the technique mere months after the incident in China. The Russian Ministry of Health expressly cited the WHO position on HHGE when implementing its inherited regime,^222^ likely seeking to position itself as a responsible actor in line with the current global consensus on the advanced ART. Ukraine, with an ART regime similar to Russia’s, and Greece have faced comparatively little political scrutiny from the international community over their decisions to allow MRTs to proceed.^223^ Absent the strong political influence and threats to their reputations seen in China and Russia, Greece and Ukraine do not appear to have altered their implementation of inherited regulation following news of their regulatees’ MRT activities.

Third, two of the cases involved enforcement for violations of inherited regulatory norms, each adopting a different enforcement style. Enforcement approaches can vary along several dimensions, including along a spectrum from persuasion and capacity-building to coercive and deterrent sanctions, with more responsive styles beginning with softer approaches and escalating if compliance is not achieved.^224^ The US FDA arguably took a more responsive approach to enforcement with the New Hope Fertility Center, sending the clinic a letter informing them of their violations and intimating greater repercussions would follow if their MRT activities continued.^225^ Officials in China took a more coercive and deterrent approach, responding with criminal prosecution and lifetime bans on performing ART and receiving public research funding for Dr He and colleagues.^226^

These examples illustrate how different styles of enforcement are possible after regulators achieve coverage with an inherited regime, providing another potential source of flexibility in the use of existing regimes for emerging technologies. In terms of effectiveness, both enforcement styles succeeded in halting and deterring undesired advanced ART activity in their respective jurisdictions.^227^ Measuring perceived legitimacy outcomes from enforcement is more challenging and may require further empirical analysis, although neither case of enforcement was met with strong public opposition. Especially if regulated entities are unaware of inherited regimes applicable to their use of an emerging technology, more responsive approaches to enforcement should be expected to yield improved legitimacy results by first seeking to inform and assist unaware regulatees before proceeding to harsher sanctions. However, as Parker notes, these responsive enforcement approaches require public support to be successful,^228^ so regulators should monitor public sentiments toward various types of emerging reproductive technologies as they proceed. Additionally, the perceived severity of the violation and political context could certainly adjust this legitimacy calculus as well, as a coercive and deterrent style of enforcement in China following the use of a highly controversial advanced ART may have improved the perceived legitimacy of regulators rather than detracted from it.^229^

These conclusions from observations of inherited regulation in action suggest that scholars and analysts should broaden their scope of inquiry to build more complete assessments of how an inherited regime performs and the political dynamics involved. While attention to inherited regulation in the past has highlighted the role of pre-existing standards and their fitness,^230^ the cases here suggest that differences in communication, monitoring, and enforcement activities and the motivations of regulatees have the potential to create divergent policy outcomes as well. Similarly, domestic and global politics may influence not only the (re)interpretation of inherited standards but also their enforcement, as illustrated by the prosecution of Dr He and colleagues against the backdrop international political pressure. Future empirical investigation on the roles and attitudes of individual regulatory bodies and officials, and the relational dynamics between regulators and regulatees as inherited regimes are implemented, may shed more light on how implementation and compliance behaviors mediate policy outcomes for inherited regulation.^231^

## VI. CONCLUSION AND IMPLICATIONS

This article has demonstrated how two rapidly emerging advanced ARTs are not truly ‘unregulated’ and reviewed case studies of several jurisdictions that have already applied existing regulatory tools to MRT and HHGE techniques. Greece, Russia, and the USA have experienced reasonable success in using inherited regulation to effectively achieve policy goals for advanced ARTs while retaining perceived legitimacy. China effectively achieved its goals, although its implementation may have suffered from diminished perceived predictability, while Ukraine appears to have struggled to attain robust, transparent regulation despite coverage with an existing regime. Comparing and evaluating these responses illustrate both the potential utility and challenges of leveraging existing regulatory regimes and offer lessons to other jurisdictions for managing advanced ARTs.

With advanced ARTs beginning to rouse global policy debates, stakeholders and policymakers should be cognizant of inherited regulation as a policy tool to achieve normative goals for managing advanced ARTs. This could be especially true in jurisdictions with limited political appetite to craft new regulatory systems. While the United Kingdom’s moves to deploy a novel regulatory framework is commendable, only a handful of jurisdictions such as Australia and Singapore appear likely to follow this approach in the short term.^232^ As seen above in Ukraine, proposed legislation to alter the governance of advanced ARTs may fail to gain enough support to become law, although it may be notable that this proposal would have authorized MRTs without providing technical standards or guidance for the techniques.^233^ While it is impossible to determine why the Ukrainian legislature did not enact the proposal without adequate political and cultural context, this outcome may reflect a coming trend of inadequate political appetite for legislative moves to govern advanced ARTs. We anticipate that defaulting to existing regulatory frameworks shall be the most likely outcome in many jurisdictions, at least initially. As such, wielding existing regimes appropriately and tailoring their requirements to the new context of advanced ARTs could become a critical governance mechanism around the world.

Adopting an inherited regulatory approach to advanced ARTs can also provide opportunities for international cooperation or transnational coordination in managing this emerging space. The Russia case with HHGE illustrates how global institutions such as the WHO can influence how states extend their existing oversight regimes and could play a capacity-building or orchestrating role in the future, if desired by states. In 2019, the WHO signaled its interest in coordinating, or supporting the coordination of, national-level regulatory responses to HHGE and could perform a similar role for other advanced ARTs.^234^ The USA taking enforcement actions for MRT activities falling outside of Mexico’s legal jurisdiction also hints at a potential for states to cooperate and coordinate how they will implement inherited regulation, even if they mobilize different types of regimes, to achieve shared goals such as managing medical tourism. Although the USA and Mexico did not actively cooperate in this case, its outcome suggests that coordinating inherited regulation could find greater use for moving forward in governing complex advanced ART activities that may involve multiple jurisdictions.

This article has also argued that some types of existing regulatory regimes may facilitate better outcomes, while others may require greater adjustments to meet the governance challenges presented by advanced ARTs. All four types of inherited regimes were able to achieve coverage of advanced ARTs, although the depth of regulation enabled by each regime type and the fitness of their goals and assumptions for advanced ARTs varied widely. Perhaps, unsurprisingly, those jurisdictions with pre-existing and robust ART regulatory regimes offered the highest fitness once coverage was achieved, as these regimes’ normative goals and regulatory tools provided high capacity to identify and manage the benefits, risks, and uncertainties of emerging reproductive technologies. Notably, some policy recommendations for emerging reproductive technologies such as longitudinal studies of health outcomes,^235^ potentially into the second generation or beyond, may be most possible under ART regimes, when accounting for cost and privacy issues. However, less-comprehensive ART oversight systems or other types of inherited regimes may require more adjustments to ensure desirable normative outcomes around advanced ARTs.

This study holds several implications for the theory of inherited regulation as well,^236^ which may merit further research. Achieving coverage of an emerging technology through pre-existing frameworks should not be taken as an inevitable result but rather as a political process that actors in a regulatory environment navigate. Domestic, regional, international, or transnational actors may become involved in the process of extending an existing regime to an emerging technology, although how these political processes evolve merits further study. Regulatory inheritance can and often does involve multiple, overlapping regimes applied to emerging technologies at once, sometimes at both the national and subnational levels, which may present coordination issues and would benefit from investigation into best practices for these scenarios. Additionally, scholars and policymakers should examine not only coverage and fitness when evaluating the potential for using an existing regime but should also consider implementation strategies to maximize effectiveness and legitimacy. Good coverage and fitness in the ideal will not translate to desirable policy outcomes without taking actions to, for example, monitor activity in the emerging space or build perceived predictability for enforcing existing rules in new ways.

As advanced ARTs continue to develop and find clinical applications, or even nonmedical uses, the need for robust governance of these innovations will only grow. Inherited regulation offers one instrument and broader strategy for policymakers to consider for establishing meaningful oversight of these emerging technologies, drawing on lessons from other jurisdictions as the implementation continues and new information on advanced ARTs arise. Weighing the strengths and weaknesses of applying inherited regulation, and its political acceptability, against other available policy tools should aid decision-makers at all levels of government in selecting prudent paths forward.

## Acknowledgments

The authors gratefully acknowledge the Carnegie Corporation of New York, which generously funded our research in this article through the Andrew Carnegie Fellows Program. The authors would like to thank Professors Eleni Kosta (Tilburg Institute for Law, Technology and Society, Tilburg University) and Sida Shen (Zhengzhou University Law School) for valuable comments and suggestions on earlier drafts of this article.

## CONFLICT OF INTEREST

The authors have no conflict of interest to report.

## Funding

Carnegie Corporation of New York

## Footnotes

1 See generally, MargaretMarsh & WandaRonner, *The Pursuit of Parenthood: Reproductive Technology from Test-Tube Babies to Uterus Transplants* (2019).

2
*Id*.; see Robert G. Edwards & Patrick C. Steptoe, *Birth After the Reimplantation of a Human Embryo*, 312 Lancet 366 (1978).

3 For example, the ‘Waller Committee’ (named after its Chairman, Professor L. Waller) in Victoria, Australia was among the first legislative commissions in the world to produce major reports and policy recommendations on the topic from 1982–84, see generally, Parliament of Victoria, Rept. No. 168, Comm. to Consider the Social, Ethical and Legal Issues Arising from In Vitro Fertilization, Report on the Disposition of Embryos Produced by In Vitro Fertilization (Aug. 1984), https://www.parliament.vic.gov.au/papers/govpub/VPARL1982-85No168.pdf.

4 For an overview of the differences between incremental and seismic innovation, from a regulatory standpoint, see generally CristieFord, Innovation and the State: Finance, Regulation, and Justice (2017).

5 See Jason Barritt et al., *Cytoplasmic Transfer in Assisted Reproduction*, 7 Hum. Reprod. Update 428 (2001); Ian Wilmut et al., *Viable Offspring Derived from Fetal Adult Mammalian Cells*, 385 Nature 810 (1997). Reproductive cloning, also called somatic cell nuclear transfer (SCNT), has most likely never been used in humans.

6 See U.S. Nat’lAcad. ofMed., U.S. Nat’lAcad. ofSci. & U.K. RoyalSociety, Heritable Human Genome Editing (Sept. 3, 2020), https://www.nap.edu/catalog/25665/heritable-human-genome-editing (hereinafter, NASEM-Royal Society Report).

7 See, eg I. Glenn Cohen et al., *The Regulation of Mitochondrial Replacement Techniques Around the World*, 21 Ann. Rev. Genomics & Hum. Genetics 565 (2020); Tetsuya Ishii & Yuri Hibino, *Mitochondrial Manipulation in Fertility Clinics: Regulation and Responsibility*, 5 Repro. Biomedicine & Soc’yOnline 93 (2018); Karinne Ludlow, *The Policy and Regulatory Context of U.S., U.K., and Australian Responses to Mitochondrial Donation Governance*, 58 Jurimetrics J. 247 (2018).

8
Nuffield Council on Bioethics, *Novel Techniques for the Prevention of Mitochondrial DNA Disorders: An Ethical Review* (2012), https://www.nuffieldbioethics.org/assets/pdfs/Novel_techniques_for_the_prevention_of_mitochondrial_DNA_disorders.pdf. These techniques have variously been called ‘mitochondrial donation’, ‘human nuclear genome transfer’, and ‘three person IVF’, though we use MRT here as it has become the most widely used and accepted term.

9 Oliver M. Russell, Gráinne S. Gorman, Robert N. Lightowlers & Doug M. Turnbull, *Mitochondrial Diseases: Hope for the Future*, 181 Cell 168 (2020).

10 Arslan A. Zaidi, *Bottleneck and Selection in the Germline and Maternal Age Influence Transmission of Mitochondrial DNA in Human Pedigrees*, 116 Proc. Nat’lAcad. Sci. 25172 (2019). Some evidence suggests paternal inheritance is possible in rare cases. Shiyu Luo et al., *Biparental Inheritance of Mitochondrial DNA in Humans*, 115 Proc. Nat’lAcad. Sci. 13039 (2018).

11 Andy Greenfield et al., *Assisted Reproductive Technologies to Prevent Human Mitochondrial Disease Transmission*, 35 Nat. Biotechnol. 1059 (2017).

12 Ana Latorre-Pellicer et al., *Mitochondrial and Nuclear Matching Shapes Metabolism and Healthy Aging*, 535 Nature 561 (2016). A longitudinal study over 10 years of five primates born via MST and three of their offspring reported no significant health issues, yet the sample size was small and the study cannot rule out possible health concerns for human use. Hong Ma et al., *Germline Transmission of Donor, Maternal and Paternal mtDNA in Primates*, 36 Hum. Reprod. 493 (2021).

13 Vasileios I. Flores et al., *Segregation of Mitochondrial DNA Heteroplasmy Through a Developmental Genetic Bottleneck in Human Embryos*, 20 Nat. CellBiol. 144 (2018).

14 See John Zhang et al., *Live Birth Derived from Oocyte Spindle Transfer to Prevent Mitochondrial Disease*, 34 Reprod. BiomedicineOnline 361 (2017); Nuno Borges-Costa et al., *First Registered Pilot Trial to Validate the Safety and Effectiveness of Maternal Spindle Transfer to Overcome Infertility Associated with Poor Oocyte Quality*, 114 FertilitySterility e71 (2020).

15 Committee on the Ethical and Social Policy Considerations of Novel Techniques for Prevention of Maternal Transmission of Mitochondrial DNA Diseases, Institute of Medicine, *Mitochondrial Replacement Techniques: Ethical, Social, and Policy Considerations* 38–39 (2016), https://www.nap.edu/catalog/21871/mitochondrial-replacement-techniques-ethical-social-and-policy-considerations. See generally, eg John B Appleby, *Should Mitochondrial Donation Be Anonymous?*, 43 J. Med. & Phil.: F. Bioethics & Phil. Med. 261 (2017); Giulia Cavaliere & César Palacios-González, *Lesbian Motherhood and Mitochondrial Replacement Techniques: Reproductive Freedom and Genetic Kinship*, 44 J. Med. Ethics 835 (2018); César Palacios-González, *Does Egg Donation for Mitochondrial Replacement Techniques Generate Parental Responsibilities?*, 44 J. Med. Ethics 817 (2018); Tina Rulli, *The Mitochondrial Replacement ‘Therapy’ Myth*, 31 Bioethics 368 (2017).

16 See Françoise Baylis, *Human Nuclear Genome Transfer (So-Called Mitochondrial Replacement): Clearing the Underbrush*, 31 Bioethics 7 (2017).

17 Of course, whether it is appropriate to conceive of MRTs intended to prevent disease as conferring ‘benefits’ on the same child that would have otherwise been born through other methods remains philosophically contested. See S. Matthew Liao, *Do Mitochondrial Replacement Techniques Affect Qualitative or Numerical Identity?*, 31 Bioethics 20 (2017); Rulli, *supra* note 15.

18 Eg Borges-Costa et al., *supra* note 14. See also Int’l Soc. for StemCell Res., ISSCR Guidelines for Stem Cell Research and Clinical Translation 3.4.8.2 (May 2020), https://www.isscr.org/policy/guidelines-for-stem-cell-research-and-clinical-translation/key-topics/mitochondrial-replacement-techniques-mrt.

19 See NASEM-Royal Society Report, *supra* note 6. Several names have been assigned to this ART, such as human germline editing. This article adopts HHGE for reasons set out by the US National Academies and UK Royal Society and to distinguish these methods from MRTs, which arguably also involve ‘germline’ modifications.

20
Nuffield Council on Bioethics, GenomeEditing andHumanReproduction (2018), https://www.nuffieldbioethics.org/publications/genome-editing-and-human-reproduction.

21 Jennifer A. Doudna & Emmanuelle Charpentier, *The New Frontier of Genome Engineering with CRISPR-Cas9*, 346 Science 1077 (2014).

22 Jennifer A. Doudna, *The Promise and Challenge of Therapeutic Genome Editing,* 578 Nature 229 (2020).

23
*Id*.; Eric S. Lander et al., *Adopt a Moratorium on Heritable Genome Editing*, 567 Nature 165 (2019).

24 Gregorio Alanis-Lobato et al., *Frequent Loss of Heterozygosity in CRISPR-Cas9–Edited Early Human Embryos*, 118 Proc. Nat’lAcad. Sci. (2021), doi: 10.1073/pnas.2004832117; Daesik Kim et al., *Evaluating and Enhancing Target Specificity of Gene-Editing Nucleases and Deaminases*, 88 AnnualRev. Biochemistry 191 (2019).

25 Maryam Mehravar et al., *Mosaicism in CRISPR/Cas9-Mediated Genome Editing*, 445 DevelopmentalBiology 156 (2019).

26 See NuffieldCouncil onBioethics, *supra* note 20.

27 See, eg Eric Lander et al., *Adopt a Moratorium on Heritable Genome Editing*, 567 Nature 167 (2019).

28 See NASEM-Royal Society Report, *supra* note 6.

29 See generally HenryGreely, The End of Sex and the Future of Human Reproduction (2018).

30 See generally, The Growing Gap Between Emerging Technologies and Legal-ethical Oversight (Gary E. Marchant et al. eds., Int’l Library of Ethics, Law & Tech., Ser. No. 7, 2011).

31 The same could be said about ‘science outpacing ethics’. See Baylis, *supra* note 16, at 9–11.

32 Diana M. Bowman, *Governing Nanotechnologies: Weaving New Regulatory Webs or Patching Up the Old?*, 2 Nanoethics 179 (2008).

33 Elen Stokes, *Nanotechnology and the Products of Inherited Regulation*, 39 J.L. & Soc’y 93 (2012).

34 See EuropeanCommission, SEC(2008) 2036, Communication from the Commission to the European Parliament, the Council and the European Economic and Social Committee: Regulatory Aspects of Nanomaterials (June 17, 2008), https://eur-lex.europa.eu/legal-content/EN/TXT/?qid=1600383808955&uri=CELEX:52008DC0366.

35
*Id*. at 107–11; Elen Stokes & Diana M. Bowman, *Looking Back to the Future of Regulating New Technologies: The Cases of Nanotechnology and Synthetic Biology*, 2 Eur. J. RiskRegulation 235, 237 (2012).

36 For example, the E.U. and Australia both crafted novel, process-based regulatory regimes to cover the process of creating genetically modified organisms. See Diana M. Bowman & Graeme A. Hodge, *A Small Matter of Regulation: An International Review of Nanotechnology Regulation*, 8 Colum. Sci. & Tech. L. Rev. 1, 23–25 (2006–2007).

37 Following broad definitions of the term ‘regulation’, we do not make categorical distinctions here between regulation and prohibition but rather see both prohibition and standard-setting as different mechanisms ‘to steer the flow of events’. See John Braithwaite, *Limits on Violence; Limits on Responsive Regulatory Theory*, 36 L. & Pol’y 432, 432 (2014); see also Julia Black, *Critical Reflections on Regulation*, 27 Austl. J.L. Phil. 1 (2002).

38 David D. Friedman, *Does Technology Require New Law?*, 25 Harv. J.L. Pub. Pol’y 71, 84–85 (2001).

39 See generally, Julia Black, *Regulatory Conversations*, 29 J.L. & Soc’y 163 (2002).

40 See Stokes & Bowman, *supra* note 35, at 235, 241.

41 See Stokes, *supra* note 33.

42 David Levi-Faur, *Regulation and Regulatory Governance*, in Handbook on thePolitics ofRegulation (David Levi-Faur ed., 2011).

43
Christopher Hood, Henry Rothstein & Robert Baldwin, The Government of Risk: Understanding Risk Regulation 21–27 (2001).

44 See Black, *supra* note 37, at 4–8.

45 Peter J. May & Søren Winter, *Reconsidering Styles of Regulatory Enforcement: Patterns in Danish Agro-Environmental Inspection*, 22 Law & Pol’y 143 (2000).

46 See generally Neil Gunningham, *Strategizing Compliance and Enforcement: Responsive Regulation and Beyond*, in ExplainingCompliance: BusinessResponses toRegulation 199 (Christine Parker & Viebeke Lehmann Nielsen eds., 2011).

47 Viebeke Lehmann Nielsen & Christine Parker, *Mixed Motives: Economic, Social, and Normative Motivations in Business Compliance*, 34 Law & Pol’y 428 (2012).

48 Karinne Ludlow, *Regulation on Agricultural Genetically Modified Organisms in Australia*, 2 J. Int’lBiotechnology L. 123 (2005); Søren C. Winter & Peter J. May, *Motivation for Compliance with Environmental Regulations*, 20 J. Pol’yAnalysis & Mgmt. 675 (2001).

49 See Julia Black, *Constructing and Contesting Legitimacy and Accountability in Polycentric Regulatory Regimes*, 2 Reg. & Governance 137, 144–48 (2008); NeilGunningham, PeterGrabosky & DarrenSinclair, Smart Regulation: Designing Environmental Policy 26–27 (1998).

50 The Human Fertilisation and Embryology (Mitochondrial Donation) Regulations 2015, N. 572 (2015), https://www.legislation.gov.uk/uksi/2015/572/contents/made (accessed Aug. 11, 2020).

51 See NuffieldCouncil onBioethics, *supra* note 8; U.K. Hum. Fertilisation & EmbryologyAuthority, *Mitochondria Replacement Consultation: Advice to Government* (Mar. 2013), https://www.hfea.gov.uk/media/2618/mitochondria_replacement_consultation_-_advice_for_government.pdf; U.K. Dep’t ofHealth, *Impact Assessment: The Human Fertilisation and Embryology (Mitochondrial Donation) Regulations 2015* (Sept. 12, 2014), https://www.legislation.gov.uk/ukia/2015/138/pdfs/ukia_20150138_en.pdf. Notably, whether the degree of public engagement was appropriate remains contested. See generally, NeilStephens & RebeccaDimond, *Legalising Mitochondrial Donation: Enacting Ethical Futures in UK Biomedical Politics* (2018).

52 The Human Fertilisation and Embryology (Mitochondrial Donation) Regulations, *supra* note 50. For more context on the assumptions underlying the UK policy, see generally Ludlow, *supra* note 7.

53 U.K. Hum. Fertilisation & Embryology Authority, *Mitochondrial Donation Treatment*, https://www.hfea.gov.uk/treatments/embryo-testing-and-treatments-for-disease/mitochondrial-donation-treatment/ (accessed Jan. 20, 2021).

54 James Gallagher, *Three-Person Baby License Granted*, BBC News (Mar. 16, 2017), https://www.bbc.com/news/health-39292381.

55 Lawmakers in Australia announced in February 2021 that they would introduce legislation to authorize MRTs in the jurisdiction. See Stephanie Dalzell, *Ground-breaking Technique to Reduce Risk of Potentially Fatal Genetic Diseases Could Soon Be Legal in Australia*, ABC NewsAustralia (Feb. 2, 2021), https://www.abc.net.au/news/2021-02-03/australia-mitochondrial-donation-to-treat-genetic-disease-bill/13114862. See also, NASEM-Royal Society Report, *supra* note 6, at 572–73, 576–77.

56 See Jessica Hamzelou, *Exclusive: World’s First Baby Born with New ‘3 Parent’ Technique*, NewScientist (Sept. 27, 2016), https://www.newscientist.com/article/2107219-exclusive-worlds-first-baby-born-with-new-3-parent-technique/.

57 Zhang et al., *supra* note 14, at 362.

58 Mina Alikani et al., *First Birth Following Spindle Transfer for Mitochondrial Replacement Therapy: Hope and Trepidation*, 34 Reprod. BiomedicineOnline 333, 333 (2017).

59 Gina Kolata, *Birth of Baby With Three Parents’ DNA Marks Success for Banned Technique*, N.Y. Times (Sept. 27, 2016), https://www.nytimes.com/2016/09/28/health/birth-of-3-parent-baby-a-success-for-controversial-procedure.html.

60 See Alikani et al., *supra* note 58, at 333.

61 See Zhang et al., *supra* note 14, at 367.

62 César Palacios-González & María de Jesús Medina-Arellano, *Mitochondrial Replacement Techniques and Mexico’s Rule of Law: On the Legality of the First Maternal Spindle Transfer Case*, 4 J.L. & Biosciences 50 (2017).

63 Reglamento de La Ley General de Salud En Materia de Investigación Para La Salud (Regulation of the General Health Law Regarding Health Research). 1987. Diario Oficial de la Federación, Feb. 6, 1987, últimas reformas Apr. 2, 2014 (Mexico), http://www.diputados.gob.mx/LeyesBiblio/regley/Reg_LGS_MIS.pdf.

64 See Palacios-González & Medina-Arellano, *supra* note 62, at 61–63.

65 Tetsuya Ishii, *Mitochondrial Replacement Techniques and Mexico’s Rule of Law: On the Legality of the First Maternal Spindle Transfer Case*, 4 J.L. & Biosciences 384, 384–85, 388 (2017); César Palacios-González & María de Jesús Medina-Arellano, *Author’s Response to Peer Commentaries: Mexico’s Rule of Law and MRTs*, 4 J.L. & Biosciences 623, 626 (2017).

66 See Palacios-González & Medina-Arellano, *supra* note 62, at 64–65.

67
*Id*.

68 See Palacios-González & Medina-Arellano, *supra* note 65, at 625.

69 Mary A. Malarkey, US Food & Drug Admin., Letter to Dr John Zhang (Aug. 4, 2017), https://www.fda.gov/media/106739/download.

70 See Zhang et al., *supra* note 14, at 364.

71
Nat’lAcad. Sci. Eng’gMed., *supra* note 15, at 10–15.

72 Consolidated Appropriations Act, 2016, Pub. L. No. 114–113, 129 Stat. 2242, § 749 (2015).

73 21 U.S.C. § 355 (2020); 42 U.S.C. 262 (2020).

74 US Food & Drug Admin., *Therapeutic Cloning and Genome Modification* (Mar. 16, 2018), https://www.fda.gov/vaccines-blood-biologics/cellular-gene-therapy-products/therapeutic-cloning-and-genome-modification. Specifically, FDA would categorize MRTs as a subclass of biologics called ‘human cell, tissue, and cellular and tissue-based products’ (HCT/P) as well as a type of drug. See Malarkey, *supra* note 69, at 2–3; see generally, 21 C.F.R. § 1271 (2020).

75 See US Food & Drug Admin., *supra* note 74.

76 See generally, 21 C.F.R. §§ 312, 601 (2020). See Malarkey, *supra* note 69, at 2–3.

77 Konstantinos Kostaras, *Spindle Transfer for the Treatment of Infertility Problems Associated to Poor Egg Quality: A Pilot Trial*, ISRCTN Registry (2018), http://www.isrctn.com/ISRCTN11455145 (accessed Jan. 21, 2021).

78 Institute of Life, *Through Pioneering Clinical Research, Institute of Life IVF Center in Greece and Embryotools in Spain Achieve Global Innovation in Assisted Reproduction*, PR Newswire (Apr. 9, 2019), https://www.prnewswire.com/news-releases/through-pioneering-clinical-research-institute-of-life-ivf-center-in-greece-and-embryotools-in-spain-achieve-global-innovation-in-assisted-reproduction-300828124.html.

79 See Kostaras, *supra* note 77.

80 Borges-Costa et al., *supra* note 14. See also Institute of Life, *Fifth Baby Born in Greece Using the Maternal Spindle Transfer Method as Part of Pilot Trial Conducted by the Institute of Life and Embryotools Scientific Team*, PR Newswire (Oct. 21, 2020), https://www.prnewswire.com/news-releases/fifth-baby-born-in-greece-using-the-maternal-spindle-transfer-method-as-part-of-pilot-trial-conducted-by-the-institute-of-life-and-embryotools-scientific-team-301155984.html.

81 Regulation (EU) No 536/2014 of the European Parliament and of the Council of Apr. 16, 2014 on Clinical Trials on Medical Products for Human Use, and Repealing Directive 2001/20/EC, OJ L 158, 27.5.2014, art. 90.

82 Britta C. van Beers, *Rewriting the Human Genome, Rewriting Human Rights Law? Human Rights, Human Dignity, and Human Germline Modification in the CRISPR Era*, 7 J.L. & Biosciences (2020), doi: 10.1093/jlb/lsaa006, at 17.

83 Calum MacKellar, *Can Maternal Spindle Transfer and Pronuclear Transfer be Prohibited Under EU Legislation?*, 25 Eur. J. Health L. 57, 70–71, 73–74 (2018).

84 Directive 2004/23/EC of the European Parliament and of the Council of Mar. 31, 2004 on Setting Standards of Quality and Safety for the Donation, Procurement, Testing, Processing, Preservation, Storage and Distribution of Human Tissues and Cells, OJ L 102, 7.4.2004.

85 See Ishii & Hibino, *supra* note 7, at 96; see also MacKellar, *supra* note 83, at 72–74.

86 Charter of Fundamental Rights of the European Union art. 3, OJ C 83/02, 30.3.2010. See van Beers, *supra* note 82, at 14; see also MacKellar, *supra* note 83, at 73.

87 The Council of Europe Treaty No. 164: The Convention for the Protection of Human Rights and Dignity of the Human Being with regard to the Application of Biology and Medicine (Oviedo Convention), April 4, 1997, art. 13, available at https://www.coe.int/en/web/conventions/full-list/-/conventions/treaty/164 (accessed Jan. 20, 2021) (hereinafter, Oviedo Convention). See also, Council of Europe, *Chart of Signatures and Ratifications of Treaty 164*, https://www.coe.int/en/web/conventions/full-list/-/conventions/treaty/164/signatures?p_auth=ev44tWmh (accessed Jan. 20, 2021).

88 See Ishii & Hibino, *supra* note 7, at 104; see also, Rosamund Scott & Stephen Wilkinson, *Germline Genetic Modification and Identity: The Mitochondrial and Nuclear Genomes*, 37 Oxford J. LegalStud. 886, 896 (2017).

89 Ley 14/2006, de 26 de Mayo, Sobre Técnicas de Reproducción Humana Asistida (On Assisted Human Reproduction Techniques). Boletín Oficial del Estado n. 126, May 27, 2006, BOE-A-2006-9292 (Spain), https://www.boe.es/buscar/pdf/2006/BOE-A-2006-9292-consolidado.pdf.

90 See Ley 14/2006, *supra* note 89, at arts. 2, 13, 20. It should be noted that Spain does not have a national agency for comprehensive ART regulation.

91
*Id*. at art. 13. See also *id*. at art 3.

92 Emily Mullin, *Pregnancy Reported in the First Known Trial of ‘Three-Person IVF’ for Infertility*, StatNews (Jan. 24, 2019), https://www.statnews.com/2019/01/24/first-trial-of-three-person-ivf-for-infertility/; see Cohen et al., *supra* note 7, at 579 (‘One of the senior members of the Spanish team asserted that they would seek approval from Spanish authorities after their MST trial ends in Greece’).

93 Nόμος 3089/2002, Ιατρική υποβοήθηση στην ανθρώπινη αναπαραγωγή, ΦΕΚ 327/Α/23-12-2002 (Law 3089/2002, Medical assistance in human reproduction, Gov Gaz. 327/Α/23-12-2002), http://www.bioethics.gr/images/pdf/ENGLISH/BIOLAW/MEDICALLY_ASSISTED_REPRODUCTION/law_3089_en.pdf.

94 Nόμος 3305/2005, Εφαρμογή της ιατρικώς υποβοηθούμενης αναπαραγωγής, ΦΕΚ Α 17/A/27.01.2005 (Law 3305/2005, Application of Medically Assisted Reproduction, Gov Gaz Α 17/A/27.01.2005), arts. 16, 19–27, http://eaiya.gov.gr/wp-content/uploads/2015/03/3305_2005.pdf, as amended by Nόμος 4272/2014, Προσαρμογή στο εθνικό δίκαιο της Εκτελεστικής Οδηγίας 2012/25/ΕΕ της Επιτροπής της 9ης Οκτωβρίου 2012 για τη θέσπιση διαδικασιών ενημέρωσης σχετικά με την ανταλλαγή, μεταξύ των κρατών−μελών, ανθρώπινων οργάνων που προορίζονται για μεταμόσχευση − Pυθμίσεις για την Ψυχική Υγεία και την Ιατρικώς Υποβοηθούμενη Αναπαραγωγή και λοιπές διατάξεις, ΦΕΚ Α 145/11.07.2014 (Law 4272/2014, Transposition into national law of Implementing Directive 2012/25/EU of 9 October 2012 laying down information procedures for the exchange, between Member States, of human organs intended for transplantation—Provisions on mental health and medically assisted reproduction and other provisions, Gov Gaz Α 145/11.07.2014), Γ. Κεφάλαιο τρίτο: Pυθμίσεις για την Ιατρικώς Υποβοήθούμενη Αναπαραγωγή (Chapter 3: Arrangements for Medically Assisted Reproduction), http://eaiya.gov.gr/wp-content/uploads/2015/12/%CE%9D%CE%9F%CE%9C%CE%9F%CE%A3-4272-_14.pdf.

95 Nόμος 3305/2005, *supra* note 94, arts. 11–12.

96
*Id*., at arts. 26–27; Nόμος 3089/2002, *supra* note 93, at art. 1. See also Maria Canellopoulou-Bottis, *The Greek Law on Genetics and Genomics—An Overview and Outlook for Future Perspectives* 13 (2018), https://papers.ssrn.com/sol3/papers.cfm?abstract_id=3295632.

97
Hellenic National Bioethics Commission, Recommendation: MitochondrialReplacement for thePrevention ofMitochondrialDiseases (Apr. 4, 2017), http://www.bioethics.gr/images/pdf/GNOMES/RECOMMENDATION_MITOCHONDRIAL_REPLACEMENT_FINAL__ENG.pdf.

98 Grigorios Leon, Angela Papetta & Chara Spiliopoulou, *Overview of the Greek Legislation Regarding Assisted Reproduction and Comparison with the EU Legal Framework*, 23 Repro. BiomedicineOnline 820, 822 (2011).

99 See Kostaras, *supra* note 77.

100 Andy Coghlan, *First Baby Born Using 3-parent Technique to Treat Infertility*, NewScientist (Jan. 18, 2017), https://www.newscientist.com/article/2118334-first-baby-born-using-3-parent-technique-to-treat-infertility/.

101 Sara Reardon, *Reports of ‘Three-Parent Babies’ Multiply*, NatureNews (Oct. 20, 2019), https://www.nature.com/news/reports-of-three-parent-babies-multiply-1.20849.

102 See Mullin, *supra* note 92.

103 Rob Stein, *Clinic Claims Success in Making Babies With 3 Parents’ DNA*, NPR (June 6, 2018), https://www.npr.org/sections/health-shots/2018/06/06/615909572/inside-the-ukrainian-clinic-making-3-parent-babies-for-women-who-are-infertile.

104 Rob Stein, *Her Son Is One Of The Few Children To Have 3 Parents’ DNA*, NPR (June 6, 2018), https://www.npr.org/sections/health-shots/2018/06/06/616334508/her-son-is-one-of-the-few-children-to-have-3-parents.

105 Pavlo Mazur et al., *Mitochondrial Replacement Theraphy* [sic] *Give No Benefits to Patients of Advanced Maternal Age*, 113 Fertility & Sterility e193, doi: 10.1016/j.fertnstert.2019.07.623.

106 Valery Zukin, *Clinical aspects of Reproductive Nuclear Transfers*, ART WorldCongress (Oct. 11, 2019), http://art2019.cme-congresses.com/wp-content/uploads/2019/10/Zukin_ART_WorldCongress_2019.pdf. See also Stein, *supra* note 103.

107 John Zhang, *Clinical Trial for the Treatment of Infertility Due to Poor Oocyte Quality*, ISRCTN Registry (2019), http://www.isrctn.com/ISRCTN13358803 (accessed Jan. 21, 2021).

108 See generally, Olga V. Danchenko, *Surrogate Motherhood v. Adoption: Comparative Characteristics and Current Challenges*, 24 Med. Law 26 (2019), doi: 10.25040/medicallaw2019.02.026.

109 Цив ільний кодекс Украї ни (Ukraine Civil Code), art. 281 (2020), https://zakon.rada.gov.ua/laws/show/en/435-15/ed20210701/sp:head#Text; Основи законодавства Укра їни про охорону здоров’я (Fundamentals of Ukrainian Legislation on Health Care), Vidomosti Verkhovnoy Rady Ukrayny, No. 2801-XII, Nov. 19, 1992 (rev’d July 24, 2020), https://zakon.rada.gov.ua/laws/main/2801-12#Text. See Mykola Gryshchenko & Alexy Pravdyuk, *Gestational Surrogacy in Ukraine*, in Handbook ofGestationalSurrogacy: InternationalClinicalPractice andPolicyIssues 250, 250 (E. Scott Sills, ed., 2016).

110 Ministry of Health of Ukraine, Про затвердження Порядкузастосуваннядопом і жних репродуктивних технолог ій вУкра їн і (On the statement of the Order of application of auxiliary reproductive technologies in Ukraine), Order No. 787, Sept. 9, 2013 (amended Mar. 6, 2014), https://zakon.rada.gov.ua/laws/show/z1697-13#n402 (replacing the prior 2008 Order No. 771).

111
*Id*. at art. 1.4; see Gryshchenko & Pravdyuk, *supra* note 109, at 251. See also Cabinet of Ministers of Ukraine, Про затвердження Ліцензійних умов провадження господарської діяльності з медичної практики (On the statement of License Conditions of carrying out economic activity in medical practice), Resolution No. 285, Mar. 2, 2016, https://zakon.rada.gov.ua/laws/show/285-2016-%D0%BF#Text; Cabinet of Ministers of Ukraine, Про затвердження Порядку акредитації закладу охорони здоров’я (On approval of the Procedure for accreditation of a health care institution), Resolution No. 765, July 15, 1997, https://zakon.rada.gov.ua/laws/show/765-97-%D0%BF#Text.

112 The Nadiya Clinic reports being licensed by the Ministry of Health of Ukraine. See Nadiya Clinic, *Our Licenses*, https://nadiya.clinic/document/ (accessed Jan. 20, 2021).

113 Про заборону репродуктивного клонування людини (On Prohibition of Reproductive Human Cloning), Vidomosti Verkhovnoy Rady Ukrayny, No. 2231-IV, Dec. 14, 2004, https://zakon.rada.gov.ua/laws/show/en/2231-15.

114 Проект Закону про Допоміжні репродуктивні технології (Draft Law on Assisted Reproductive Technologies) art. 8, No. 8629–1, Aug. 1, 2018, http://w1.c1.rada.gov.ua/pls/zweb2/webproc4_2?pf3516=8629-1&skl=9.

115 Oviedo Convention, *supra* note 87; see also Council of Europe, *supra* note 87.

116 See Fundamentals of Ukrainian Legislation on Health Care, *supra* note 109, at arts. 44–45.

117 See, eg Ministry of Health of Ukraine, Про затвердження Порядку проведення клінічнихвипробувань лікарських засоб ів таекспертизиматер іалів клінічних випробувань і Типового положення про комісії з питань етики (On the statement of the Pro-cedure for carrying out clinical trials of medicines and examination of materials of clinical trials and the Standard position about the commissions concerning ethics), Order No. 690, Sept. 23, 2009, https://zakon.rada.gov.ua/laws/show/z1010-09#Text.

118 Evidence of the clinical trial does not appear in the Ukrainian clinical trial registry as of the time of writing. See State Expert Center of the Ministry of Health of Ukraine, Інформація щодо клінічних випробувань в Україні (Information on clinical trials in Ukraine), https://clinicaltrials.dec.gov.ua/ (accessed Jan. 21, 2021).

119 Correspondence from Mykola Chashchyn, Executive Secretary of the Commission on Bioethics, National Academy of Sciences of Ukraine, to author (Oct. 19, 2020) (on file with authors).

120 The clinical trial registry reports ethical approval on Dec. 28, 2018, while the first birth occurred in Jan. 2017. See Zhang, *supra* note 107.

121 Correspondence from Mykola Chashchyn, *supra* note 119.

122
*Id*.

123 John Zhang et al., *Pregnancy Derived from Human Nuclear Transfer*, 80 FertilitySterility 56, 56 (2003).

124 John Zhang et al., *Pregnancy Derived from Human Zygote Pronuclear Transfer in a Patient Who Had Arrested Embryos After IVF*, 33 Reprod. BiomedicineOnline 529 (2016).

125 Ministry of Health, People’s Republic of China (2003), The Technical Norms for Human Assisted Reproduction Technologies (人类辅助生殖技术规范), art. 3, § 7, http://www.nhc.gov.cn/bgt/pw10303/200708/68ba58984aba4a44a3bcf74b0c3e2048.shtml; Ministry of Health, People’s Republic of China (2003), Human Assisted Reproductive Technology and Human Sperm Bank Ethical Principles (人类辅助生殖技术和人类精子库伦理原则), art. 3, § 7, http://www.nhc.gov.cn/bgt/pw10303/200708/68ba58984aba4a44a3bcf74b0c3e2048.shtml.

126 See Ishii, *supra* note 65, at 388.

127 The Technical Norms for Human Assisted Reproduction Technologies, *supra* note 125, at art. 3, § 9.

128 Tetsuya Ishii, *Potential Impact of Human Mitochondrial Replacement on Global Policy Regarding Germline Gene Modification*, 29 Repro. BiomedicineOnline 150, 154 (2014). See also AchimRosemann, LiJiang & XinqingZhang, NuffieldCouncil onBioethics, *The Regulatory and Legal Situation of Human Embryo, Gamete and Germ Line Gene Editing Research and Clinical Applications in the People’s Republic of China* 9–10 (May 2017), https://www.nuffieldbioethics.org/assets/pdfs/GEHR-background-paper-gene-editing-in-China.pdf.

129 See Rosemann, Jiang & Zhang, *supra* note 128, at 17.

130
*Id*.

131
Nuffield Council on Bioethics, *supra* note 20, at 111–112.

132 These services may include surrogacy or sex selection. *Id*. F. Bai & D.Y. Wang et al., *Assisted Reproductive Technology Service Availability, Efficacy and Safety in Mainland China: 2016*, 35 Hum. Reprod. 1477 (2020).

133 Zhonghua Renmin Gongheguo Zhiye Yishi Fa (中华人民共和国执业医师法) (Law of the People’s Republic of China on Medical Practitioners) (promulgated by the Nat’l People’s Cong., June 26, 1998, effective May 1, 1999), art. 13–16, http://www.npc.gov.cn/zgrdw/englishnpc/Law/2007-12/11/content_1383574.htm.

134 Antonio Regalado, *Exclusive: Chinese Scientists Are Creating CRISPR Babies*, MIT Tech. Rev. (Nov. 25, 2018), https://www.technologyreview.com/2018/11/25/138962/exclusive-chinese-scientists-are-creating-crispr-babies/.

135 Dennis Normile, *CRISPR Bombshell: Chinese Researcher Claims to Have Created Gene-Edited Twins*, Science (Nov. 26, 2018), https://www.sciencemag.org/news/2018/11/crispr-bombshell-chinese-researcher-claims-have-created-gene-edited-twins.

136 David Cyranoski & Heidi Ledford, *Genome-edited Baby Claim Provokes International Outcry*, NatureNews (Nov. 26, 2018), https://www.nature.com/articles/d41586-018-07545-0.

137 See, eg Henry T. Greely, *CRISPR’d Babies: Human Germline Genome Editing in the ‘He Jiankui Affair’*, 6 J.L. & Biosciences 111, 151–69 (2019).

138 Jon Cohen, *Did CRISPR Help—or Harm—the First-Ever Gene-Edited Babies?*, NatureNews (Aug. 1, 2019), https://www.sciencemag.org/news/2019/08/did-crispr-help-or-harm-first-ever-gene-edited-babies.

139 Sharon Begley, *Ethical Issues Plagued Newly Surfaced Paper by ‘CRISPR Babies’ Scientist*, StatNews (Dec. 10, 2018), https://www.statnews.com/2018/12/10/crispr-babies-scientists-paper-rejected/; Antonio Regalado, *China’s CRISPR Babies: Read Exclusive Excerpts from the Unseen Original Research*, MIT Tech. Rev. (Dec. 3, 2019), https://www.technologyreview.com/2019/12/03/131752/chinas-crispr-babies-read-exclusive-excerpts-he-jiankui-paper/.

140 Chen Wang, Xiaomei Zhai, Xinqing Zhang, Limin Li, Jianwei Wang & De-pei Liu, *Gene-Edited Babies: Chinese Academy of Medical Sciences’ Response and Action*, 393 Lancet 25, 26 (2019).

141 See Preetika Rana, *How a Chinese Scientist Broke the Rules to Create the First Gene-Edited Babies*, WallStreet J. (May 10, 2019), https://www.wsj.com/articles/how-a-chinese-scientist-broke-the-rules-to-create-the-first-gene-edited-babies-11557506697. But see Jane Qiu, *Chinese Government Funding May Have Been Used for ‘CRISPR Babies’ Project, Documents Suggest*, StatNews (Feb. 25, 2019), https://www.statnews.com/2019/02/25/crispr-babies-study-china-government-funding/.

142 ‘基因 编辑婴儿’案一审宣判 贺建奎等三被告人被追究刑事责任 (He Jiankui and Other Three Defendants Were Prosecuted for Criminal Responsibility), XinhuaNet (Dec. 30, 2019), http://www.xinhuanet.com/2019-12/30/c_1125403802.htm.

143 J. Benjamin Hurlbut, *Imperatives of Governance: Human Genome Editing and the Problem of Progress*, 63 PerspectivesBiology & Med. 177, 186 (2020).

144 ‘China Focus: Three Jailed in China’s “Gene-Edited Babies” Trial’, XinhuaNet (Dec. 30, 2019), http://www.xinhuanet.com/english/2019-12/30/c_138667350.htm.

145
*Id*.

146 Zhonghua Renmin Gongheguo Xingfa (中华人民共和国刑法) (Criminal Law of the People’s Republic of China) (promulgated by the Nat’l People’s Cong., Jul. 1, 1979, rev’d Mar. 14, 1997, effective Oct. 1, 1997), art. 336, http://www.npc.gov.cn/zgrdw/englishnpc/Law/2007-12/13/content_1384075.htm.

147 Ruipeng Lei & Renzong Qiu, *Chinese Bioethicists: He Jiankui’s Crime is More than Illegal Medical Practice*, HastingsCenter (Jan. 4, 2020), https://www.thehastingscenter.org/chinese-bioethicists-he-jiankuis-crime-is-more-than-illegal-medical-practice/.

148
*Id*.

149 David Cyranoski, *China Set to Introduce Gene-Editing Regulation Following CRISPR-Baby Furore*, NatureNews (May 20, 2019), https://www.nature.com/articles/d41586-019-01580-1.

150 中华人民共和国刑法修正案(十一)[Amendment to the Ciminal Law of the People’s Republic of China (XI)] (promulgated by the Nat’l People’s Cong., Dec. 27, 2020, effective Mar. 1, 2021), http://www.gov.cn/xinwen/2020-12/27/content_5573660.htm.

151 David Cyranoski, *Russian Biologist Plans More CRISPR-Edited Babies*, NatureNews (June 10, 2019), https://www.nature.com/articles/d41586-019-01770-x.

152 See, eg Editorial, *Act Now on CRISPR Babies*, 570 Nature 137 (2019).

153 Jon Cohen, *Deaf Couple May Edit Embryo’s DNA to Correct Hearing Mutation*, Science (Oct. 21, 2019), https://www.sciencemag.org/news/2019/10/deaf-couple-may-edit-embryo-s-dna-correct-hearing-mutation. This view of deafness, however, depends on a contested view of disability with significant ethical and social implications for the Deaf community and society writ large. Robert Sparrow, *Implants and Ethnocide: Learning from the Cochlear Implant Controversy*, 25 Disability & Soc’y 455 (2010).

154 Cyranoski, *supra* note 151.

155 Об основах охраны здоровья граждан в Российской Федерации (On the Fundamentals of Protecting the Health of Citizens in the Russian Federation), 2011, No. 323-FZ, art. 55, https://minzdrav.gov.ru/documents/7025-federalnyy-zakon-ot-21-noyabrya-2011-g-323-fz-ob-osnovah-o.

156 Resolution of the Government of the Russian Federation, April 16, 2012, No. 291, О лицензировании медицинской деятельности (On the Licensing of Medical Activities), https://roszdravnadzor.gov.ru/medactivities/licensing/documents/35817.

157 Ministry of Health of the Russian Federation, Order of August 30, 2012 No. 107n, о порядке использования вспомогательных репродуктивных технологий, противопоказаниях и ограничениях к их применению (On the Use of Assisted Reproductive Technologies: Contraindications and Limitations to their Use), http://www.medkirov.ru/docs/id/4C543B.

158 Tetsuya Ishii, *Germline Genome Editing in Clinics: The Approaches, Objectives and Global Society*, 16 BriefingsFunctionalGenomics 46 (2017).

159
*Id*.; RumianaYotova, NuffieldCouncil onBioethics, TheRegulation ofGenomeEditing andHumanReproductionUnderInternationalLaw, EU Law andComparativeLaw 49–50 (June 2017), https://www.nuffieldbioethics.org/wp-content/uploads/Report-regulation-GEHR-for-web.pdf.

160 Olga Dobrovidova, *Calling Embryo Editing ‘Premature’, Russian Authorities Seek to Ease Fears of a Scientist Going Rogue*, StatNews (Oct. 16, 2019), https://www.statnews.com/2019/10/16/russia-health-ministry-calls-human-embryo-editing-premature/.

161
*Id*.

162 World Health Org. Expert Advisory Comm., *As We Explore Options for Global Governance, Caution Must Be Our Watchword* (Nov. 8, 2019), https://www.who.int/ethics/topics/human-genome-editing/ethics-explore-options-for-global-governance.pdf?ua=1.

163 Jon Cohen, *Embattled Russian Scientist Sharpens Plans to Create Gene-Edited Babies*, Science (Oct. 21, 2019), https://www.sciencemag.org/news/2019/10/embattled-russian-scientist-sharpens-plans-create-gene-edited-babies.

164 David Cyranoski, *Russian ‘CRISPR-Baby’ Scientist Has Started Editing Genes in Human Eggs with Goal of Altering Deaf Gene*, NatureNews (Oct. 18, 2019), https://www.nature.com/articles/d41586-019-03018-0.

165 See Stokes, *supra* note 33; Stokes & Bowman, *supra* note 35.

166 Further, why and precisely how governments elect to use existing regimes to govern emerging technologies is a significant question, though this article will focus on the normative dimensions and outcomes of defaulting to inherited systems. See Alberto Asquer & Inna Krachkovskaya, *Uncertainty, Institutions and Regulatory Responses to Emerging Technologies: CRISPR Gene Editing in the US and the EU (2012–2019)*, Reg. & Governance (2020), doi: http://doi.org/10.1111/rego.12335.

167 See generally, Epistemology andMethodology ofComparativeLaw (Mark Van Hoecke ed., 2005).

168 For example, a 2019 survey of over 1500 respondents primarily from the USA, United Kingdom, Canada, and Australia revealed higher support than previously reported for using HHGE for medical reasons among some demographics, particularly among male respondents. See Abbie Jedwab, Danya F. Vears, Cheryl Tse & Christopher Gyngell, *Genetics Experience Impacts Attitudes Towards Germline Gene Editing: A Survey of Over 1500 Members of the Public*, 65 J. Hum. Genetics 1055–65 (2020).

169 See Stokes & Bowman, *supra* note 35.

170 Mullin, *supra* note 92.

171
Xinhua Net, *supra* note 142.

172 See Dobrovidova, *supra* note 160.

173 While ART or products regulatory regimes may include criminal sanctions, we refer here to the extension of more general criminal law into the advanced ART space.

174
Xinhua Net, *supra* note 144.

175 Palacios-González & Medina-Arellano, *supra* note 62, at 64–65.

176 Palacios-González & Medina-Arellano, *supra* note 62.

177 Correspondence from Mykola Chashchyn, *supra* note 119.

178 Stein, *supra* note 103.

179 US Food & Drug Admin., *supra* note 74.

180 MacKellar, *supra* note 83, 70–71; van Beers, *supra* note 82, at 16–17.

181 See also Cohen et al., *supra* note 7, at 580.

182 See Stein, *supra* note 104.

183 Mullin, *supra* note 92.

184 See Black, *supra* note 39.

185 See Stephens & Dimond, *supra* note 51.

186
Hellenic National Bioethics Commission, *supra* note 97.

187 See Cohen, *supra* note 163.

188 See Dobrovidova, *supra* note 160.

189 See Stokes, *supra* note 33.

190 See Stokes, *supra* note 33.

191 See Stokes & Bowman, *supra* note 35.

192 See generally EmilyJackson, Regulating Reproduction: Law, Technology and Autonomy (2001).

193 Nόμος 3305/2005, *supra* note 94, arts. 1, 6; Human Assisted Reproductive Technology and Human Sperm Bank Ethical Principles, *supra* note 125; Ley 14/2006, *supra* note 89, art. 3.

194 Fundamentals of Ukrainian Legislation on Health Care, *supra* note 109, art. 48; On the Fundamentals of Protecting the Health of Citizens in the Russian Federation, *supra* note 155 art. 55; Ukraine Civil Code, *supra* note 109, art. 281.

195 See Karinne Ludlow, *Genetic Identity Concerns in the Regulation of Novel Reproductive Technologies*, 7 J.L. & Biosciences (2020), doi: 10.1093/jlb/lsaa004, at 3–4.

196 See eg World Medical Association, *The Declaration of Helsinki*, last amended Oct. 2013, https://www.wma.net/policies-post/wma-declaration-of-helsinki-ethical-principles-for-medical-research-involving-human-subjects/.

197 Of course, social and cultural context mediate how these principles are constructed and applied. See Tuija Takala, *What Is Wrong with Global Bioethics? On the Limitations of the Four Principles Approach*, 10 Cambridge Q. HealthcareEthics 72 (2001).

198 Correspondence from Mykola Chashchyn, *supra* note 119.

199 Ishii, *supra* note 65, at 384–85.

200 See Daniel Carpenter, Justin Grimmer & Eric Lomazoff, *Approval Regulation and Endogenous Consumer Confidence: Theory and Analogies to Licensing, Safety, and Financial Regulation*, 4 Reg. & Governance 383, 386, 400–01 (2010); Stokes, *supra* note 33, at 94–95, 112.

201 US Food & Drug Admin., *supra* note 74.

202 See Nicola Lacey, *Criminalisation as Regulation: The Role of Criminal Law*, in RegulatingLaw 144 (Christine Parker et al. eds., 2004).

203 Criminal Law of the People’s Republic of China, *supra* note 146, arts. 1–2; see generally, Susan Trevaskes, *The Shifting Sands of Punishment in China in the Era of ‘Harmonious Society’*, 32 Law & Pol’y 332 (2010).

204 Lacey, *supra* note 202.

205 Lei & Qiu, *supra* note 147. However, the Chinese criminal code has now been updated through new legislation to directly address HHGE. See Amendment to the Criminal Law of the People’s Republic of China (XI), *supra* note 150.

206 Palacios-González & Medina-Arellano, *supra* note 62, at 64–65.

207 See Lei & Qiu, *supra* note 147.

208 Inherited regimes in other jurisdictions contain similar instruments. For example, Australian law currently prohibits MRTs and HHGE, although lawmakers are considering novel legislation to permit MRTs in a controlled setting. Ludlow, *supra* note 7, 247, 255; see Dalzell, *supra* note 55.

209 See generally Jody Freeman & Jim Rossi, *Agency Coordination in Shared Regulatory Space*, 125.
Harv. L. Rev. 1131 (2012).

210 On designing policy mixes generally, see generally Gunningham, Grabosky & Sinclair, *supra* note 49.

211 Stokes & Bowman, *supra* note 35.

212 See Ludlow, *supra* note 48; Nielsen & Parker, *supra* note 47; Winter & May, *supra* note 48. See generally Valerie Braithwaite, *Dancing with Tax Authorities: Motivational Postures and Non-compliant Actions*, in TaxingDemocracy (Valerie Braithwaite ed., 2003).

213 Cohen, *supra* note 163.

214 Correspondence from Mykola Chashchyn, *supra* note 119.

215 See Normile, *supra* note 135; *but see* Qiu, *supra* note 141.

216 Eg Sharon Begley & Andrew Joseph, *The CRISPR Shocker: How Genome-Editing Scientist He Jiankui Rose from Obscurity to Stun the World*, StatNews (Dec. 17, 2018), https://www.statnews.com/2018/12/17/crispr-shocker-genome-editing-scientist-he-jiankui/.

217 See Malarkey, *supra* note 69; Joel Achenbach, *Ethicists Approve ‘3 Parent’ Embryos to Stop Diseases, But Congressional Ban Remains*, Wash. Post (Feb. 3, 2016), https://www.washingtonpost.com/news/speaking-of-science/wp/2016/02/03/to-prevent-disease-ethicists-approve-creation-of-embryos-with-three-genetic-parents/.

218 Malarkey, *supra* note 69.

219
*Id*.

220 César Palacios-González, *Mexico and Mitochondrial Replacement Techniques: What a Mess*, 128 BritishMed. Bull. 97, 104 (2018).

221 Hurlbut, *supra* note 143, at 186–87.

222 Dobrovidova, *supra* note 160.

223 Of course, this lower level of scrutiny may be, in part, a result of the United Kingdom already legislating on MRTs or the general perception that MRTs are less controversial than HHGE.

224 See Gunningham, *supra* note 46; Bridget M. Hutter, *Variations in Regulatory Enforcement Styles*, 11 Law & Pol’y 153 (1989); May & Winter, *supra* note 45.

225 Malarkey, *supra* note 69.

226
Xinhua Net, *supra* note 142.

227 At the time of writing, no further reports of HHGE in China or MRTs in the USA have been reported.

228 Christine Parker, *The ‘Compliance’ Trap: The Moral Message in Responsive Regulatory Enforcement*, 40 Law & Pol’y 591 (2006).

229 Hurlbut, *supra* note 143, at 186–87.

230 See Stokes, *supra* note 33; Stokes & Bowman, *supra* note 35.

231 See, eg Anat Gofen, *Mind the Gap: Dimensions and Influence of Street-Level Divergence*, 24 J. Pub. Admin. Res. & Theory 473 (2014); Gordon Peake & Miranda Forsyth, *Street-Level Bureaucrats in a Relational State: The Case of Bougainville*, Pub. Admin. & Dev. (2021), doi: 10.1002/pad.1911.

232 See Cohen et al., *supra* note 7, at 572–73, 576–77.

233 Draft Law on Assisted Reproductive Technologies, *supra* note 114.

234 See World Health Org. Expert Advisory Comm., *supra* note 162. In 2021, the WHO advanced this goal further by releasing twin reports providing policy options and recommendations for national and global governance of HHGE. See WorldHealthOrg. ExpertAdvisoryComm., *Human Genome Editing: A Framework for Governance* (2021), https://www.who.int/publications/i/item/9789240030060;*World Health Org. Expert Advisory Comm., Human Genome Editing: Recommendations* (2021), https://www.who.int/publications/i/item/9789240030381.

235 See NASEM-Royal Society Report, *supra* note 6; NuffieldCouncil onBioethics, *supra* note 8.

236 See Stokes, *supra* note 33; Stokes & Bowman, *supra* note 35.

